# Supplementary material for: An analogue of the Prolactin Releasing Peptide reduces obesity and promotes adult neurogenesis
Source: EMBO Rep. 2023 Dec 20;25(1):351–77. doi: 10.1038/s44319-023-00016-2 (PMC10897398; doi:10.1038/s44319-023-00016-2)
Supplement: Supplementary file 1 — Appendix with title page [file 44319_2023_16_MOESM1_ESM.pdf]

Appendix Figures (Jorgensen et al., 2023): Table of Content

Name	Page	Description
Appendix Figure S1	1	LiPR does not affect astrogliogenesis in the MBH
Appendix Figure S2	2	Additional RNAseq data and confirmation of RNAseq by RT-qPCR
Appendix Figure S3	3	Additional data from calcium imaging of hiPSC-derived hypothal. neurons
Appendix Figure 4S	4	A schematic of LiPR effects on hypothalamic adult neurogenesis

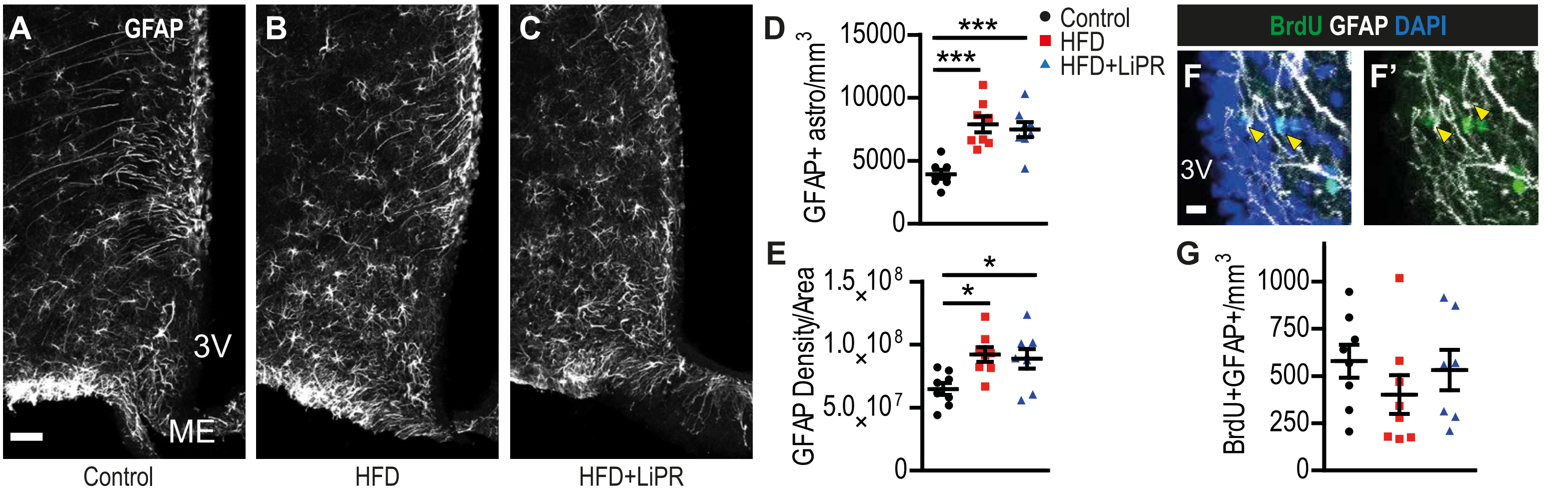

Appendix Figure S1 – LiPR does not affect astrogliogenesis in the MBH

(A-C) Representative confocal images of HVZ stained for GFAP in Control (A), HFD (B) nd HFD + LiPR (C) of 4mo HFD group.

(D-E) Quantification of GFAP+ astrocytes (D) and GFAP density (E) in MBH parenchyma of 4mo HFD group.

(F) Representative confocal image of BrdU+ astrocytes in MBH parenchyma.

(G) Quantification of BrdU+ astrocytes in MBH parenchyma.

Scale bars: 50 µm (A-C), 20 µm (F). n = 8.

Data information: One-Way ANOVA (panel D: $F(2,21) = 16.22$, $p < 0.0001$; panel E: $F(2,21) = 5.67$, $p = 0.011$).

*$p < 0.05$, ***$p < 0.001$ (Bonferroni's test). Data are presented as mean ± SEM.

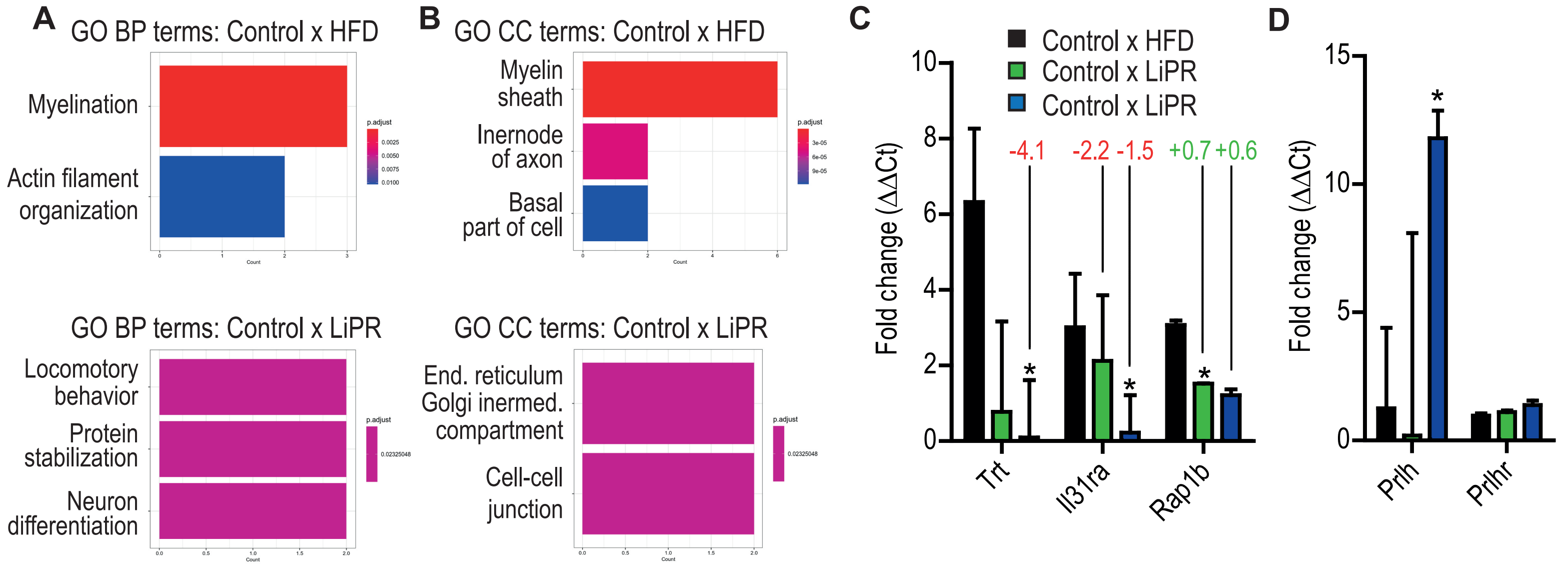

Appendix Figure S2 – Additional RNAseq data and confirmation of RNAseq by RT-qPCR
(A) Bulk MBH RNAseq GO terms for Biological Processes (BP) for Control Diet vs HFD (top graph) and Control Diet vs Control Diet+LiPR (bottom graph).
(B) Bulk MBH RNAseq GO terms for Cell Compartments (CC).
(C) Relative mRNA fold change of Trt, Il31ra and Rap1b compared to Gapdh in cDNA used for the bulk RNAseq of the MBH. Numbers above the bar graphs represent expression change values for a given gene from the RNAseq data.
(D) Relative mRNA fold change of Prlh and Prlhr compared to Gapdh in cDNA used for the bulk RNAseq of the MBH. Data information: un-paired two-tailed T-Test, *$p < 0.05$. Data are presented as mean ± SEM.

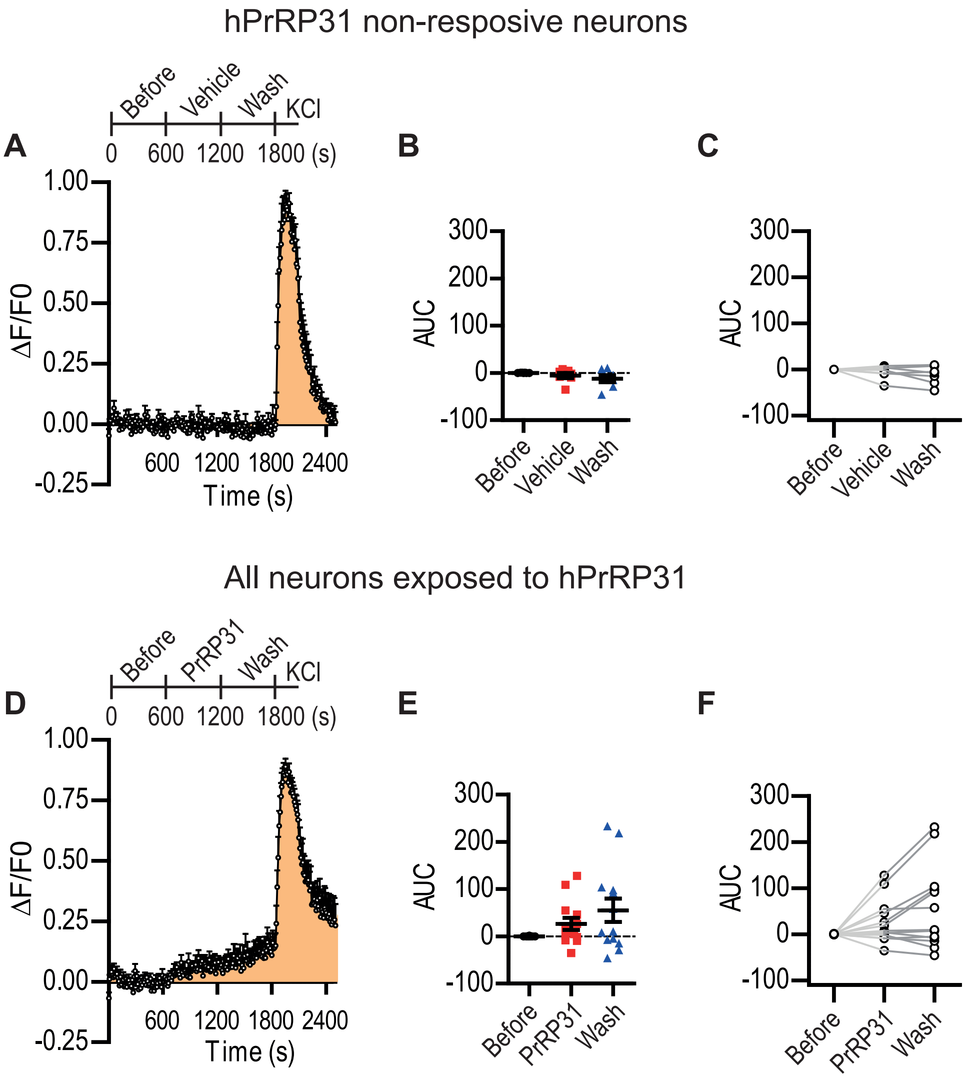

Appendix Figure S3 – Additional data from calcium imaging of hiPSC-derived hypothalamic neurons
(A,D) A graph of the relative fluorescence change (ΔF/F0) of Rhod-3 as a function of time before and during hPrRP31 application in medium, wash-out and KCl positive control in hiPSC-derived neurons non-responsive to hPrRP31 (A) and all neurons exposed to hPrRP31.
(B,E) A summary of fluorescence before (0-600 s), during hPrRP31 (600-1200 s) or wash-out (1200-1800 s) in Area Under Curve (AUC) for hPrRP31 non-responsive (B) and all neurons exposed to hPrRP31 (E).
(C,F) Individual neurons from calcium imaging in the before-after plot from the non-responsive (C) and all exposed neurons (F).
n = 7 for non-responsive, n =13 all neurons. Data are presented as mean ± SEM.

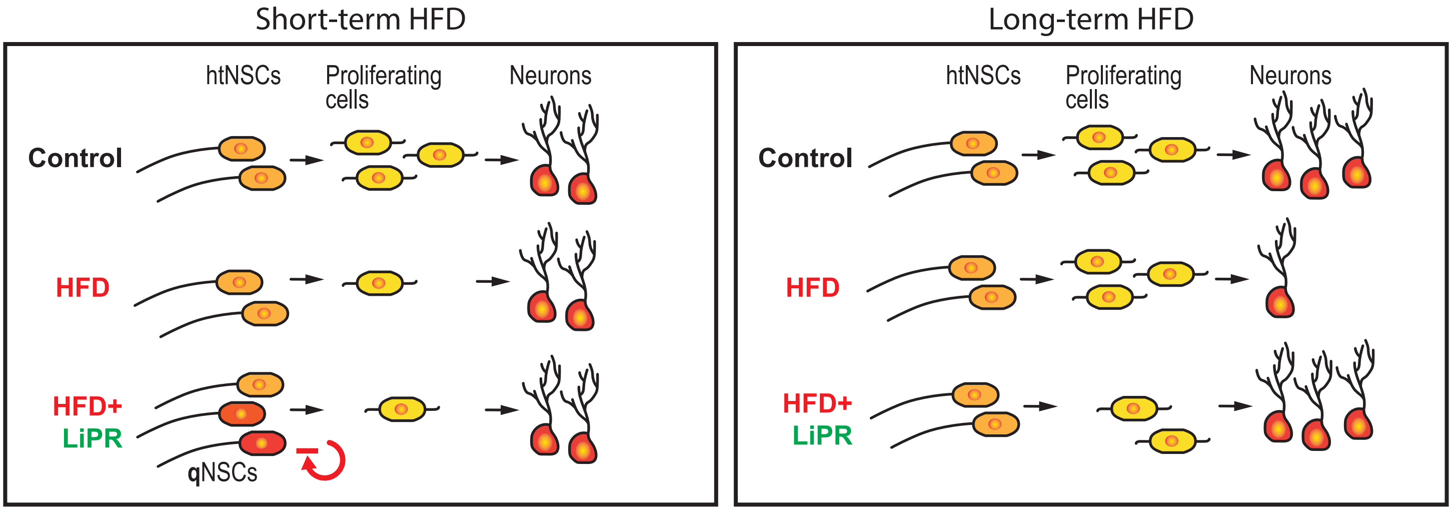

Appendix Figure S4 – A schematic of LiPR effects on hypothalamic adult neurogenesis
(Left) LiPR decreases proliferation and activation of htNSCs during short and intermediate HFD protocols.
(Right) LiPR increases survival of new hypothalamic neurons during long-term HFD and obesity.
