# Supplementary figures and images for: An analogue of the Prolactin Releasing Peptide reduces obesity and promotes adult neurogenesis
Source: EMBO Rep. 2023 Dec 20;25(1):351–77. doi: 10.1038/s44319-023-00016-2 (PMC10897398; doi:10.1038/s44319-023-00016-2)

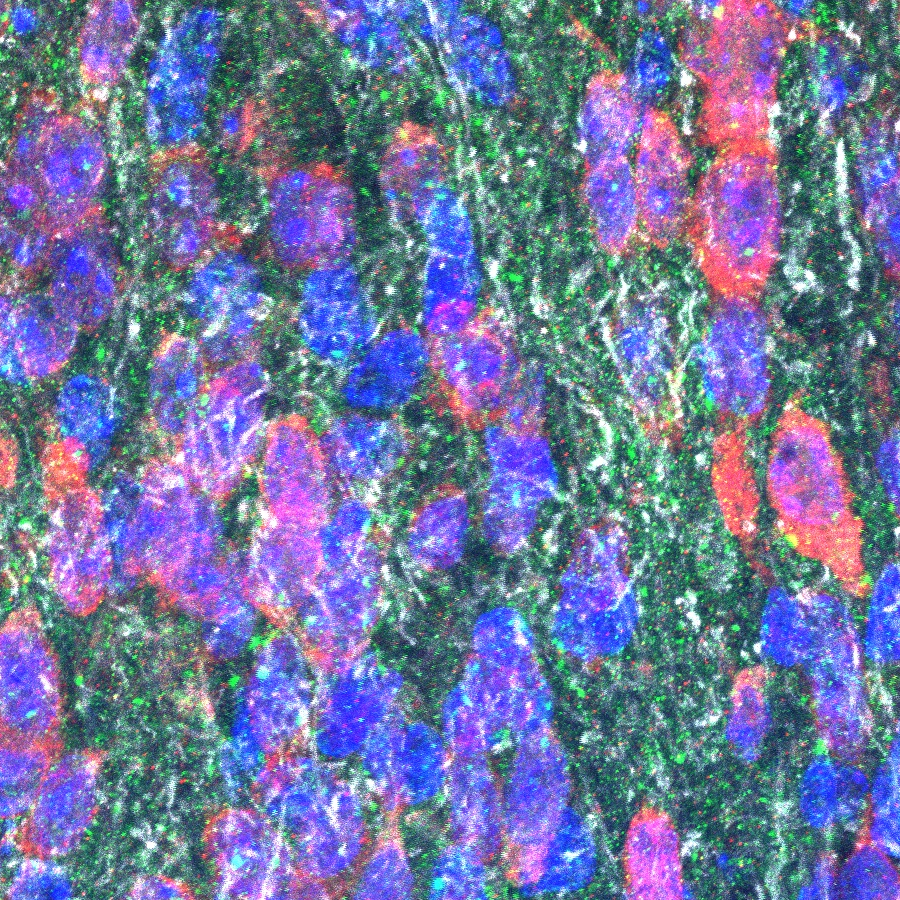

Supplement: Supplementary file 3 — Source Data Fig. 1 [file 44319_2023_16_MOESM3_ESM.zip › Fig1I picture.png]

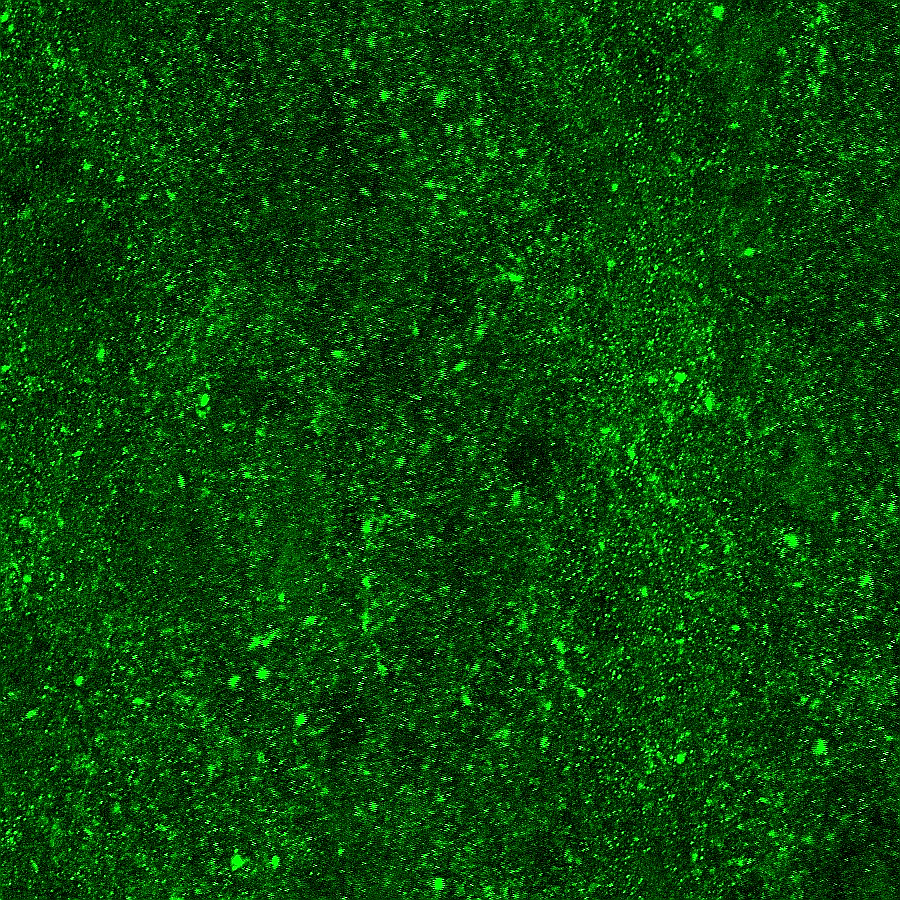

Supplement: Supplementary file 3 — Source Data Fig. 1 [file 44319_2023_16_MOESM3_ESM.zip › Fig1I' picture.tif]

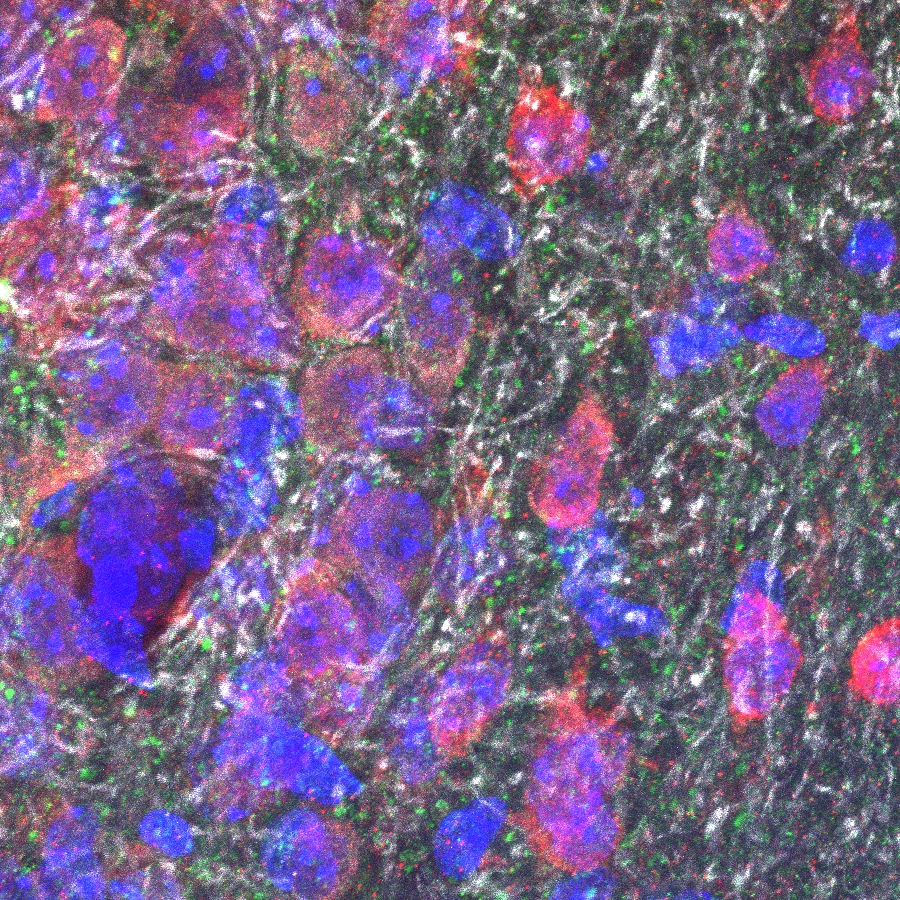

Supplement: Supplementary file 3 — Source Data Fig. 1 [file 44319_2023_16_MOESM3_ESM.zip › Fig1J picture.png]

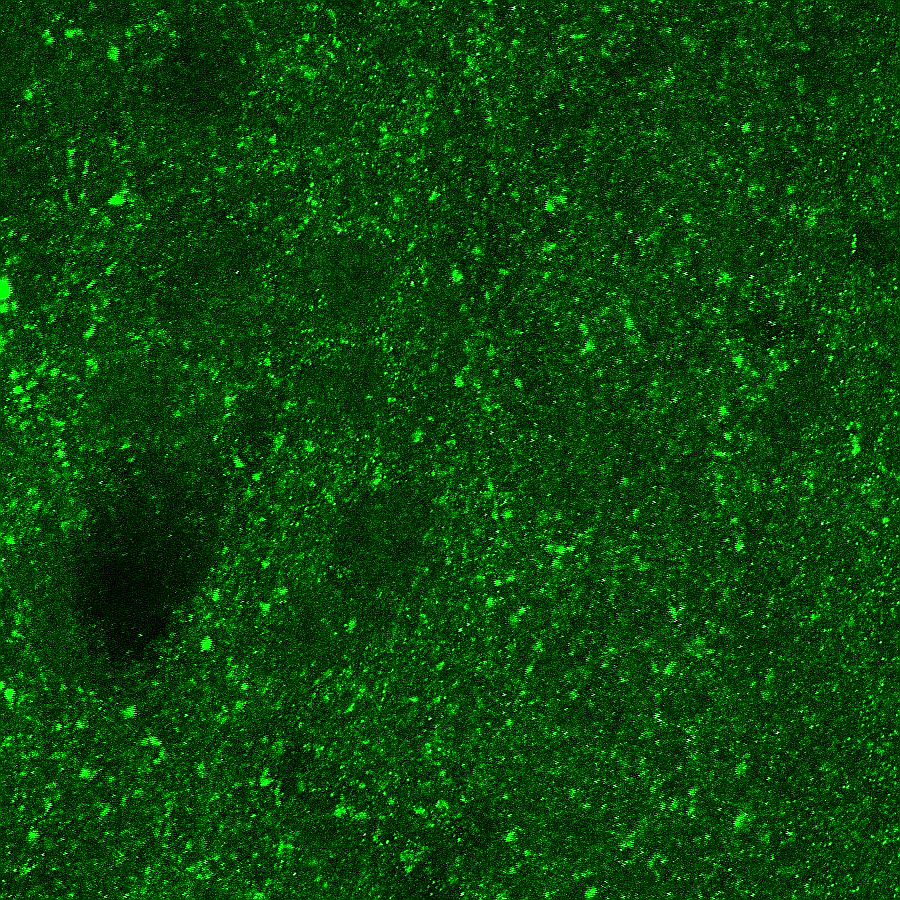

Supplement: Supplementary file 3 — Source Data Fig. 1 [file 44319_2023_16_MOESM3_ESM.zip › Fig1J' picture.png]

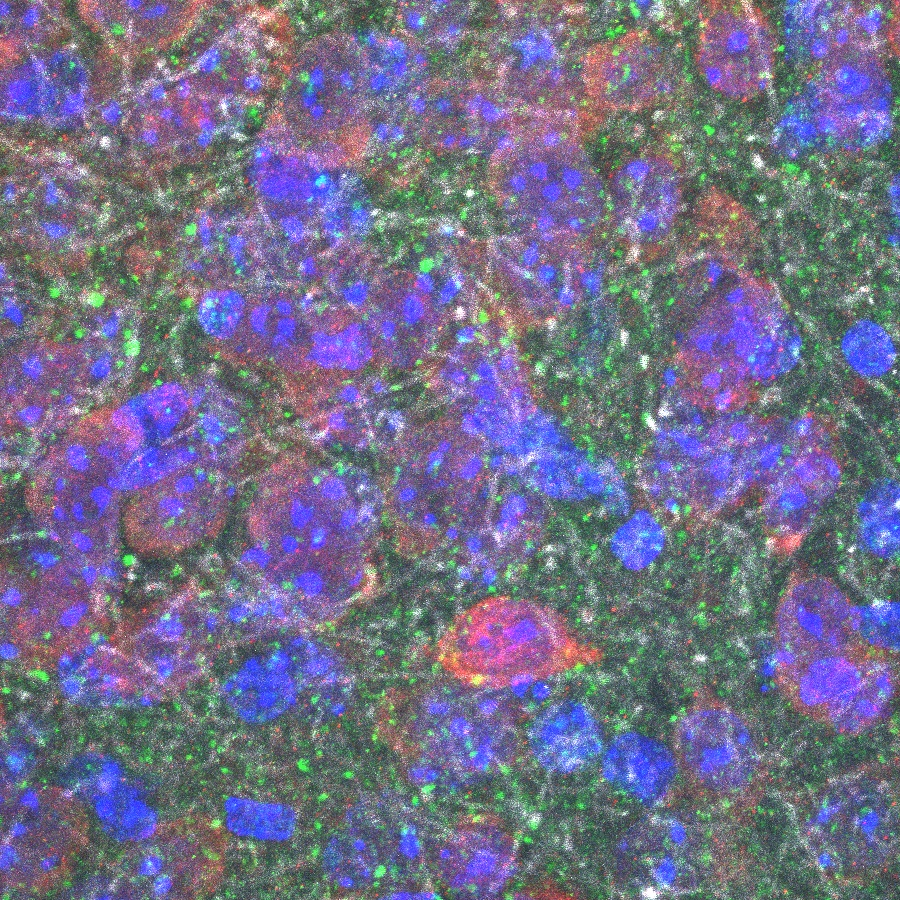

Supplement: Supplementary file 3 — Source Data Fig. 1 [file 44319_2023_16_MOESM3_ESM.zip › Fig1K picture.png]

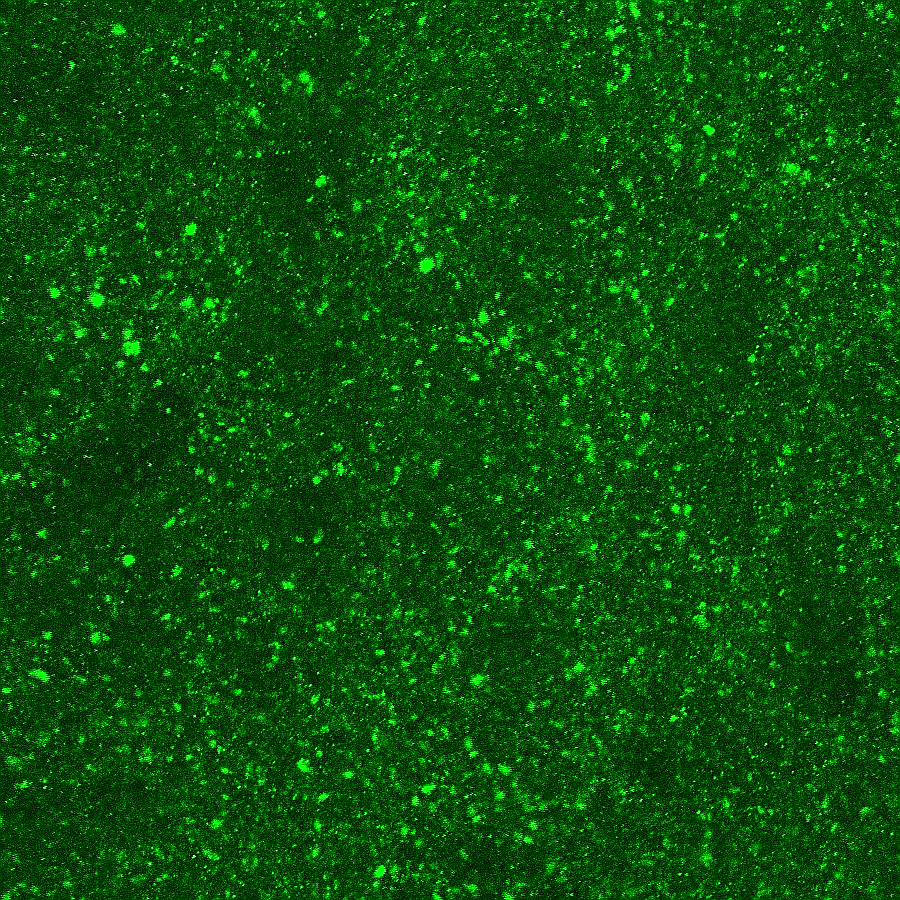

Supplement: Supplementary file 3 — Source Data Fig. 1 [file 44319_2023_16_MOESM3_ESM.zip › Fig1K' picture.png]

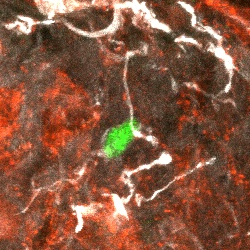

Supplement: Supplementary file 3 — Source Data Fig. 1 [file 44319_2023_16_MOESM3_ESM.zip › Fig1M picture.png]

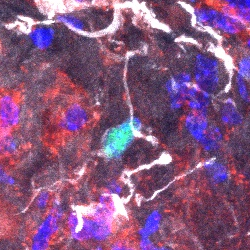

Supplement: Supplementary file 3 — Source Data Fig. 1 [file 44319_2023_16_MOESM3_ESM.zip › Fig1M' picture.png]

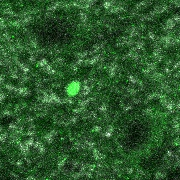

Supplement: Supplementary file 3 — Source Data Fig. 1 [file 44319_2023_16_MOESM3_ESM.zip › Fig1O picture.png]

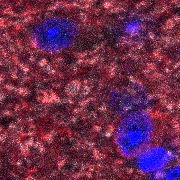

Supplement: Supplementary file 3 — Source Data Fig. 1 [file 44319_2023_16_MOESM3_ESM.zip › Fig1O' picture.png]

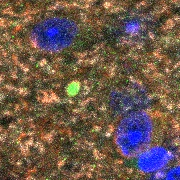

Supplement: Supplementary file 3 — Source Data Fig. 1 [file 44319_2023_16_MOESM3_ESM.zip › Fig1O'' picture.png]

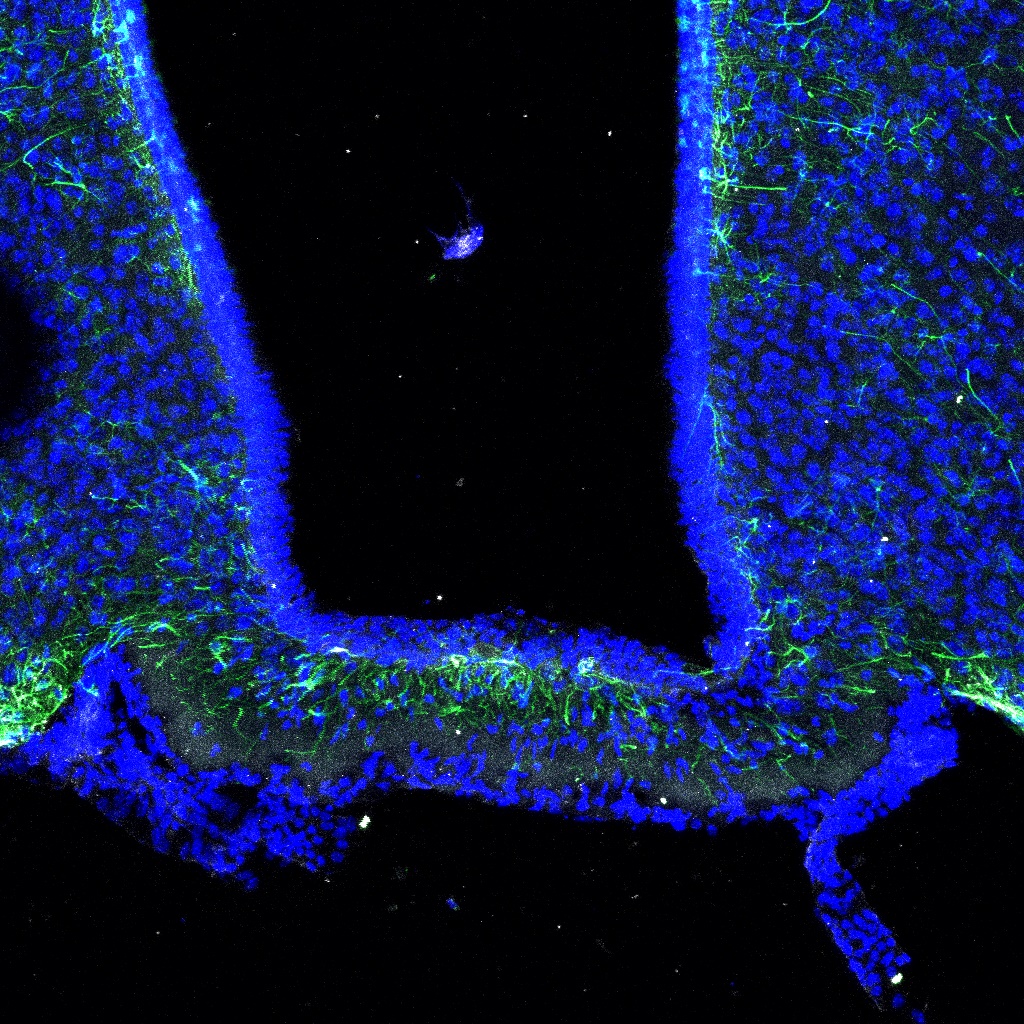

Supplement: Supplementary file 4 — Source Data Fig. 2 [file 44319_2023_16_MOESM4_ESM.zip › Fig2A picture.jpg]

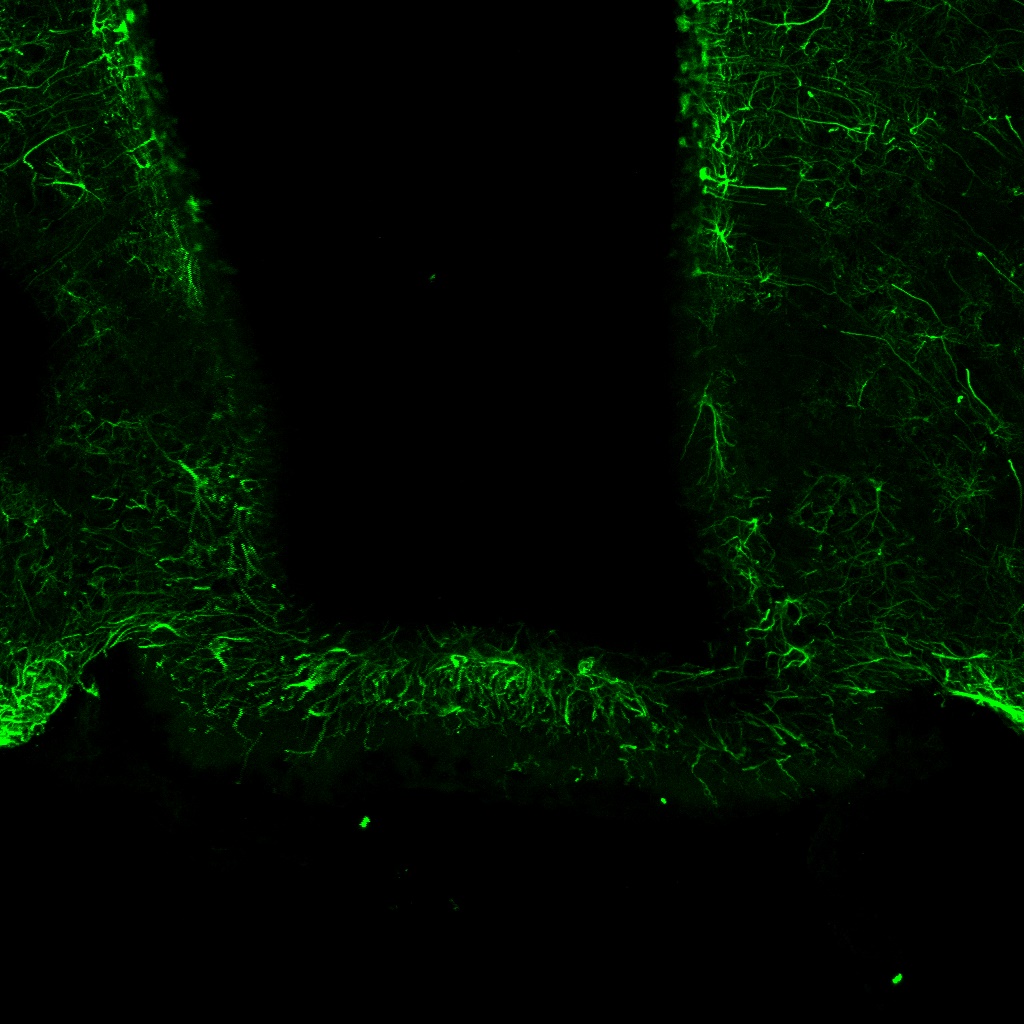

Supplement: Supplementary file 4 — Source Data Fig. 2 [file 44319_2023_16_MOESM4_ESM.zip › Fig2A' picture.jpg]

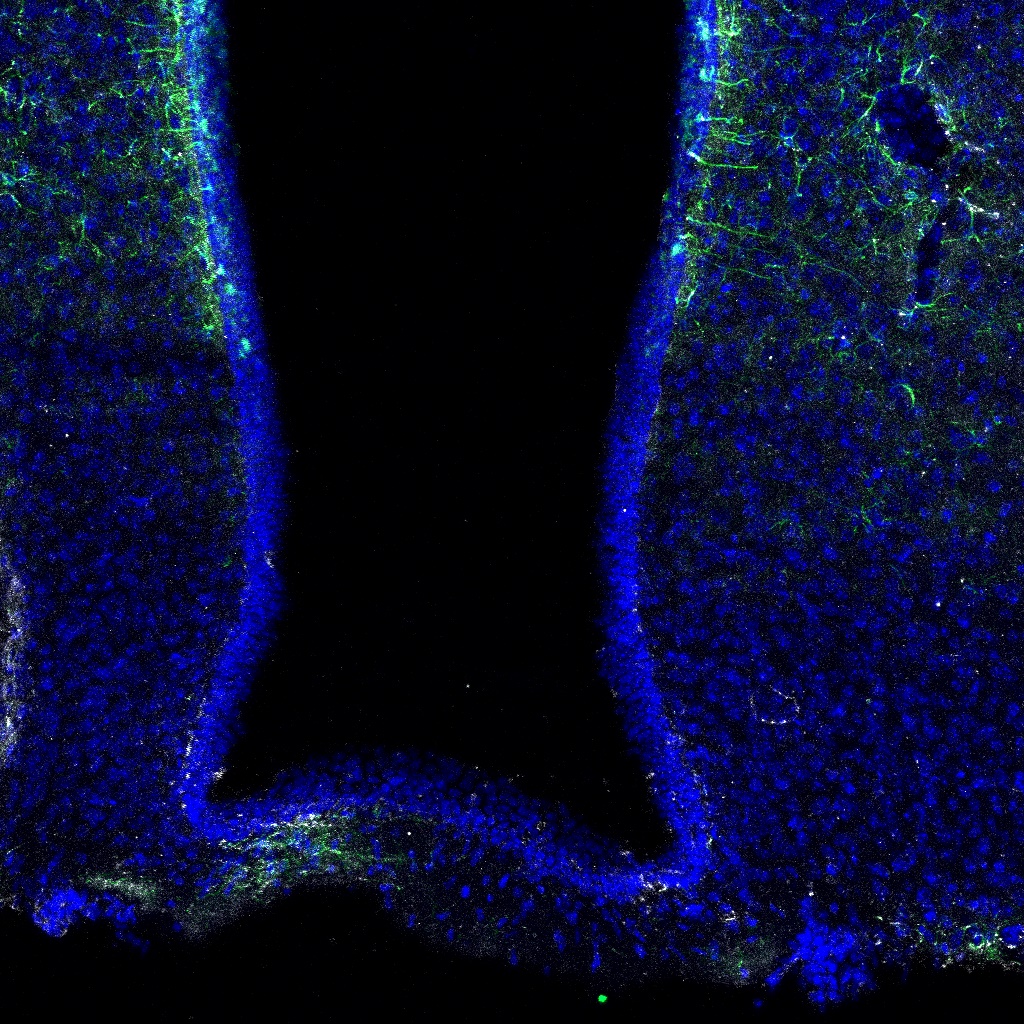

Supplement: Supplementary file 4 — Source Data Fig. 2 [file 44319_2023_16_MOESM4_ESM.zip › Fig2B picture.jpg]

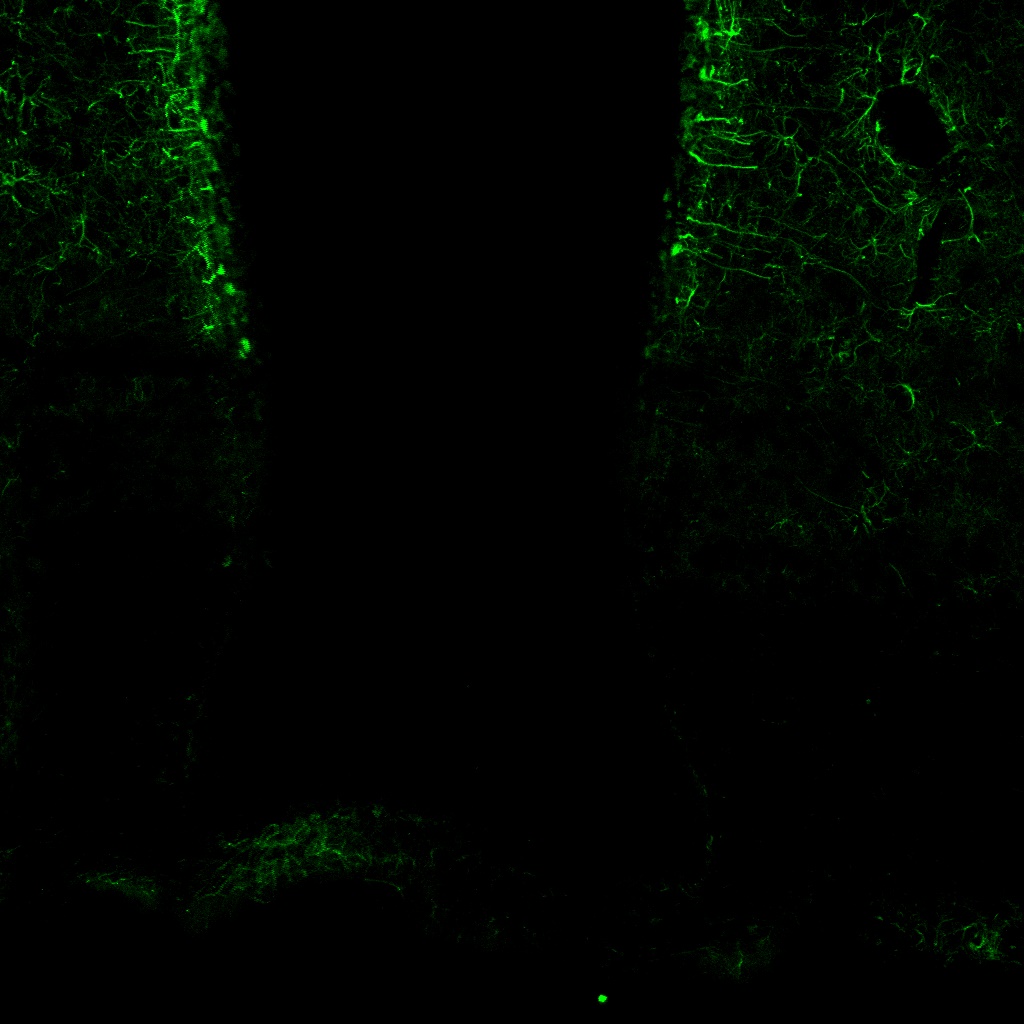

Supplement: Supplementary file 4 — Source Data Fig. 2 [file 44319_2023_16_MOESM4_ESM.zip › Fig2B' picture.jpg]

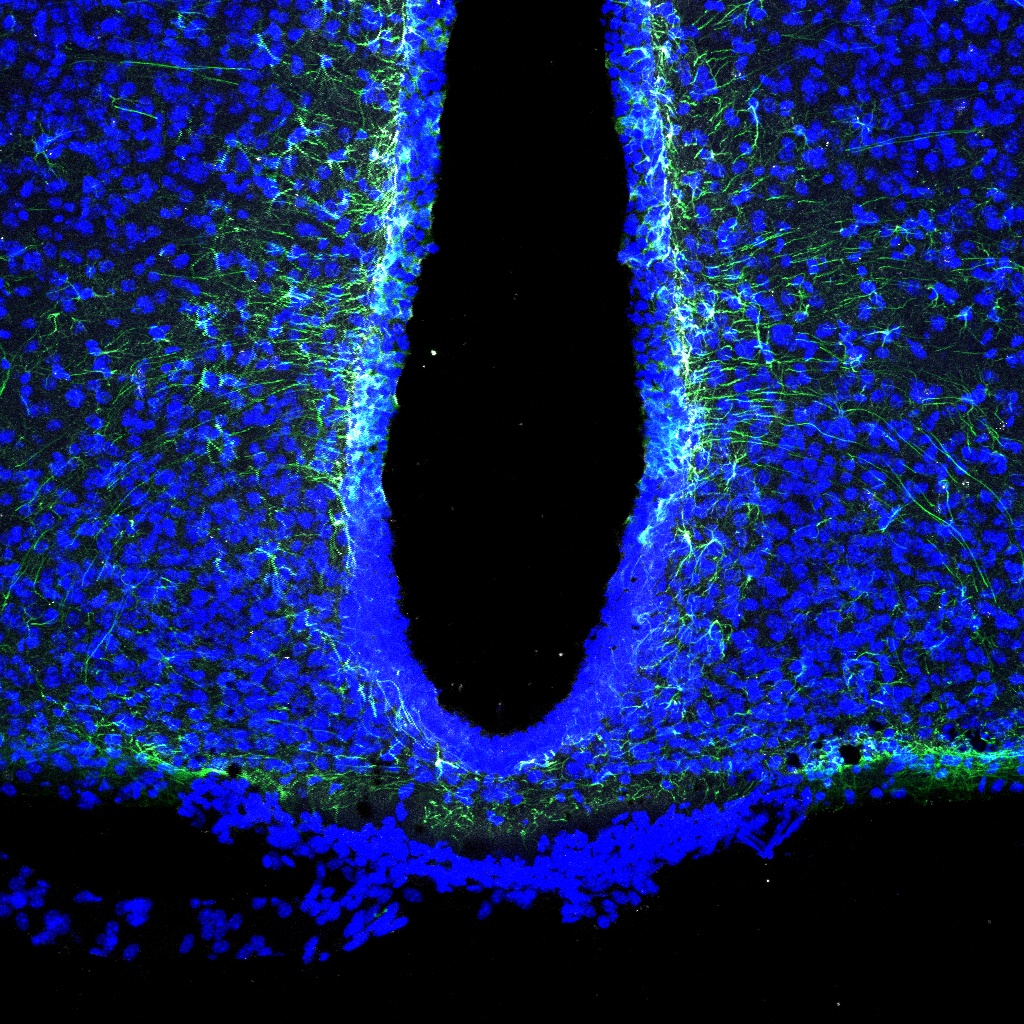

Supplement: Supplementary file 4 — Source Data Fig. 2 [file 44319_2023_16_MOESM4_ESM.zip › Fig2C picture.jpg]

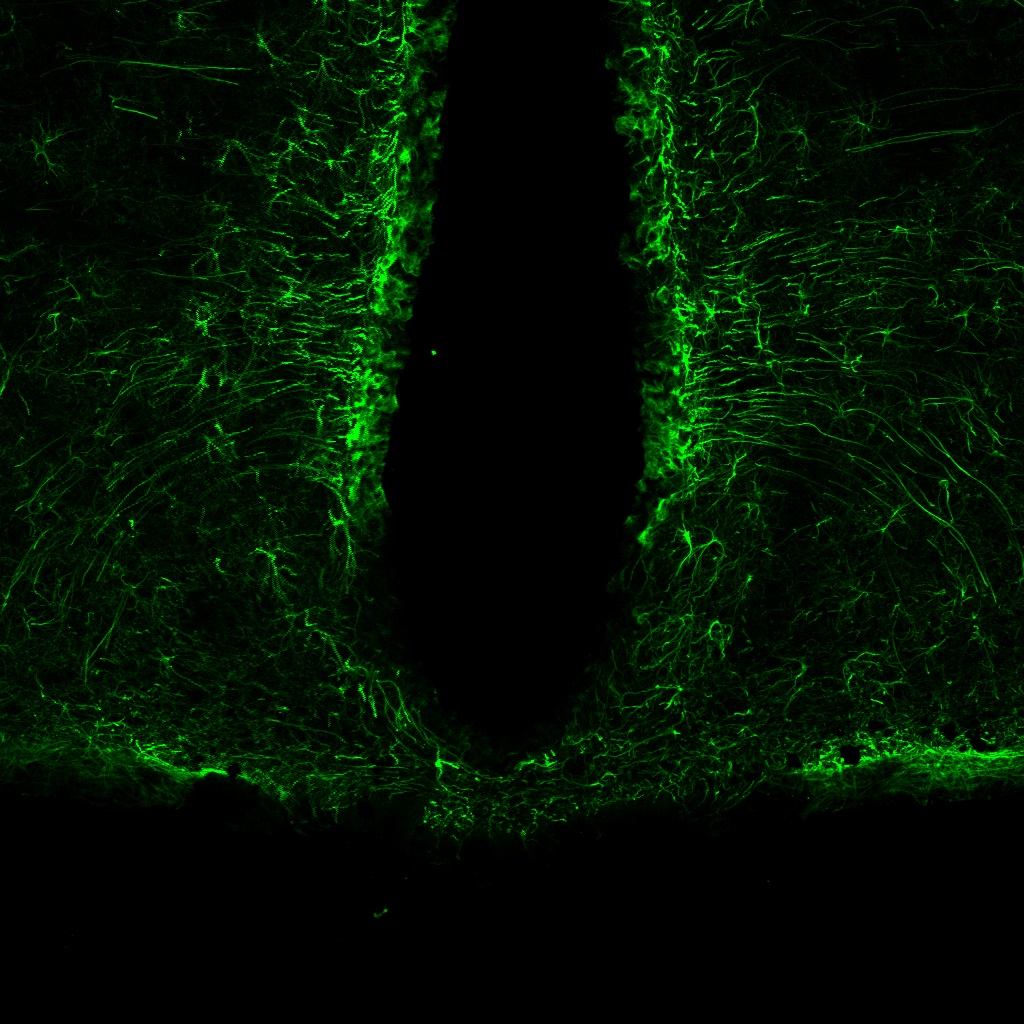

Supplement: Supplementary file 4 — Source Data Fig. 2 [file 44319_2023_16_MOESM4_ESM.zip › Fig2C' picture.jpg]

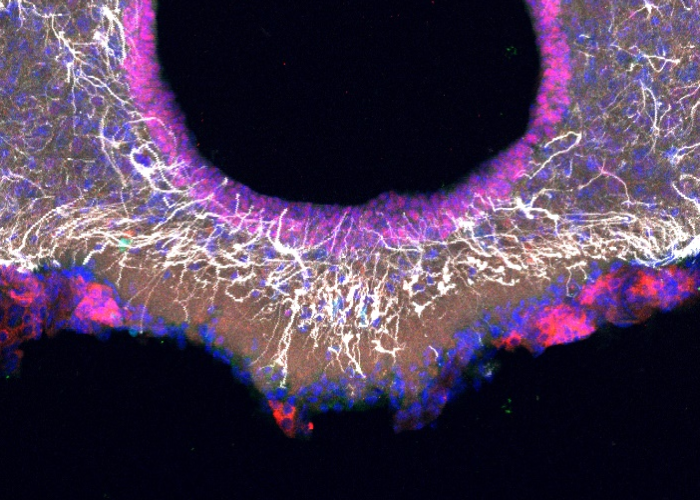

Supplement: Supplementary file 4 — Source Data Fig. 2 [file 44319_2023_16_MOESM4_ESM.zip › Fig2H picture.png]

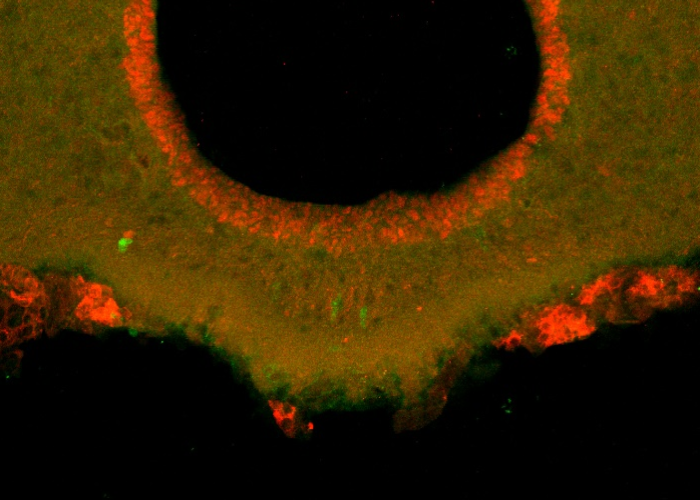

Supplement: Supplementary file 4 — Source Data Fig. 2 [file 44319_2023_16_MOESM4_ESM.zip › Fig2H' picture.png]

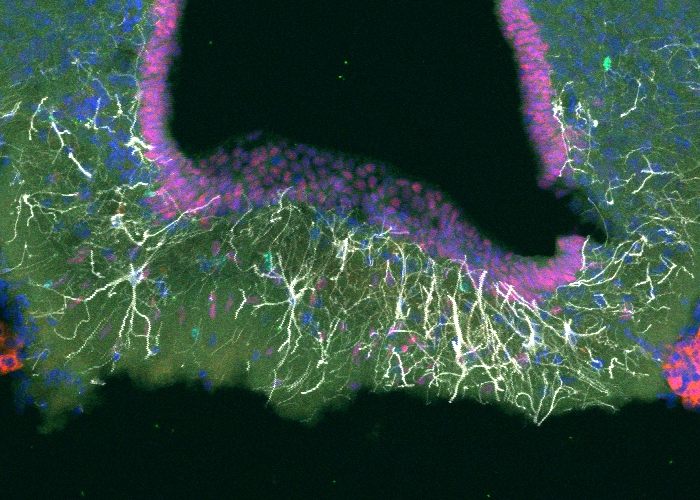

Supplement: Supplementary file 4 — Source Data Fig. 2 [file 44319_2023_16_MOESM4_ESM.zip › Fig2I picture.png]

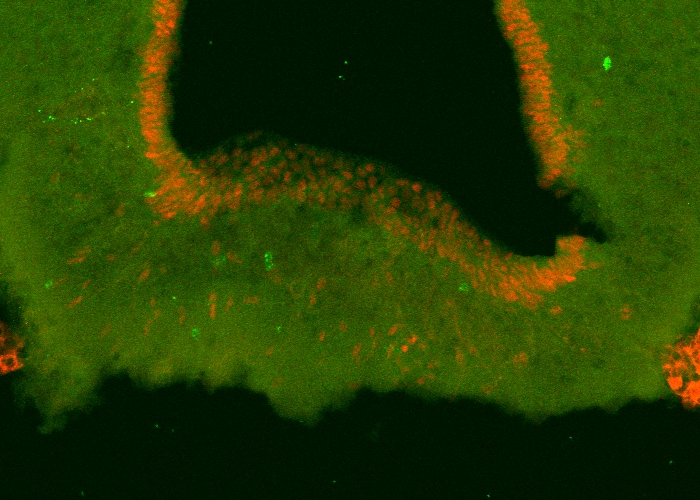

Supplement: Supplementary file 4 — Source Data Fig. 2 [file 44319_2023_16_MOESM4_ESM.zip › Fig2I' picture.png]

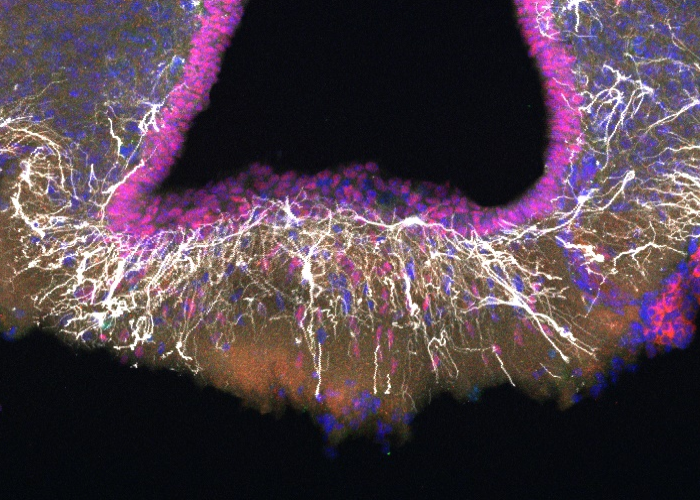

Supplement: Supplementary file 4 — Source Data Fig. 2 [file 44319_2023_16_MOESM4_ESM.zip › Fig2J picture.png]

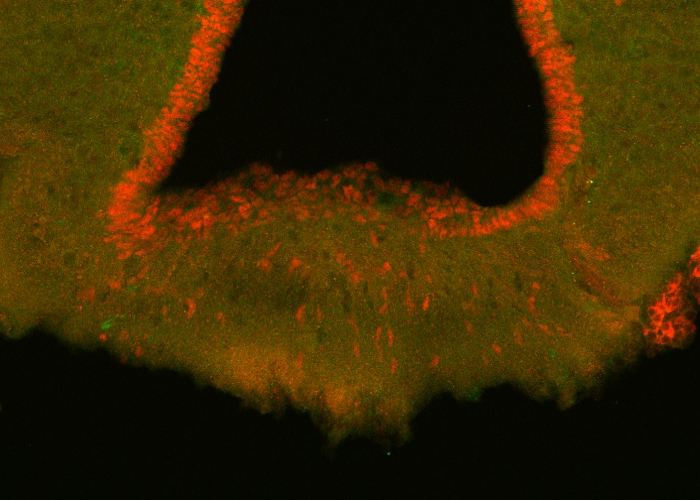

Supplement: Supplementary file 4 — Source Data Fig. 2 [file 44319_2023_16_MOESM4_ESM.zip › Fig2J' picture.png]

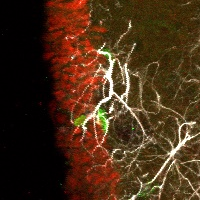

Supplement: Supplementary file 4 — Source Data Fig. 2 [file 44319_2023_16_MOESM4_ESM.zip › Fig2K picture.png]

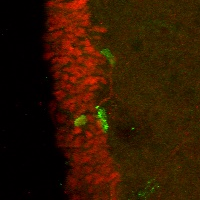

Supplement: Supplementary file 4 — Source Data Fig. 2 [file 44319_2023_16_MOESM4_ESM.zip › Fig2K' picture.png]

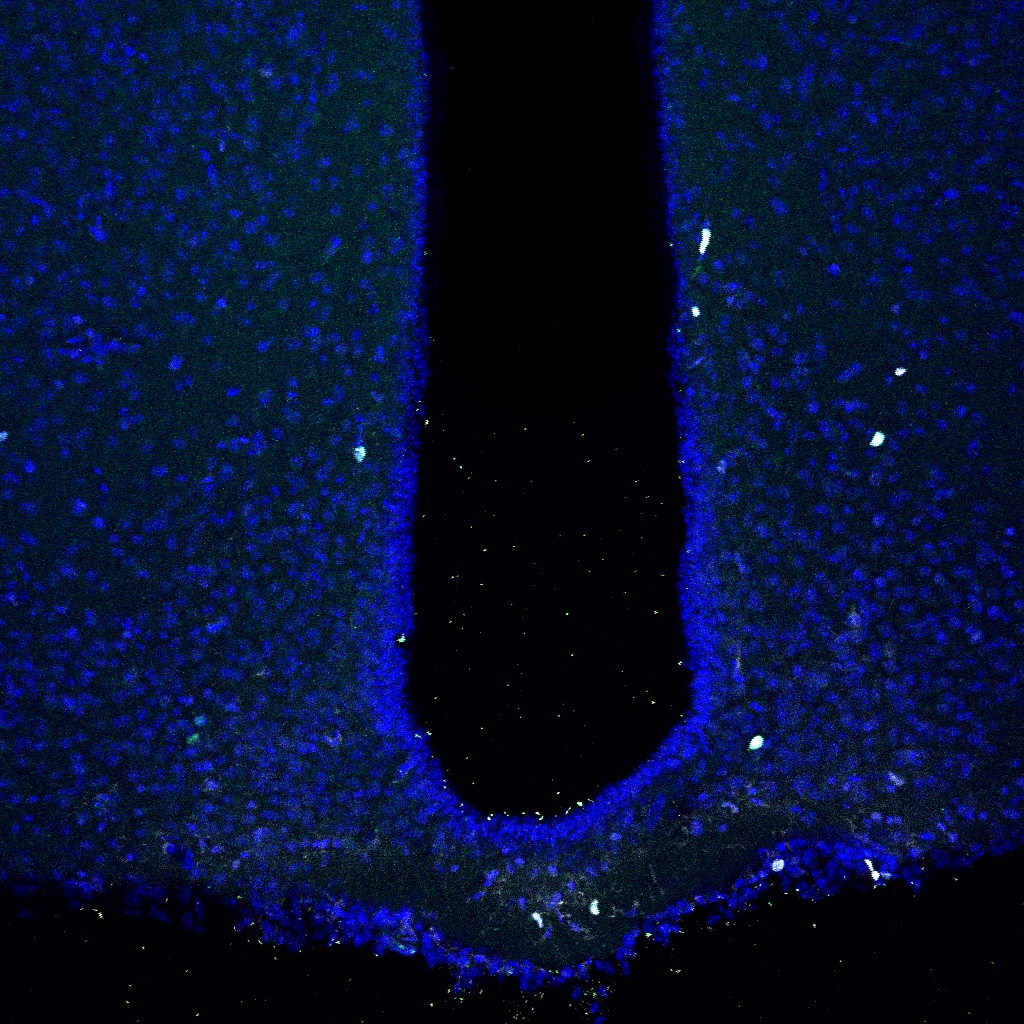

Supplement: Supplementary file 4 — Source Data Fig. 2 [file 44319_2023_16_MOESM4_ESM.zip › Fig2O picture.jpg]

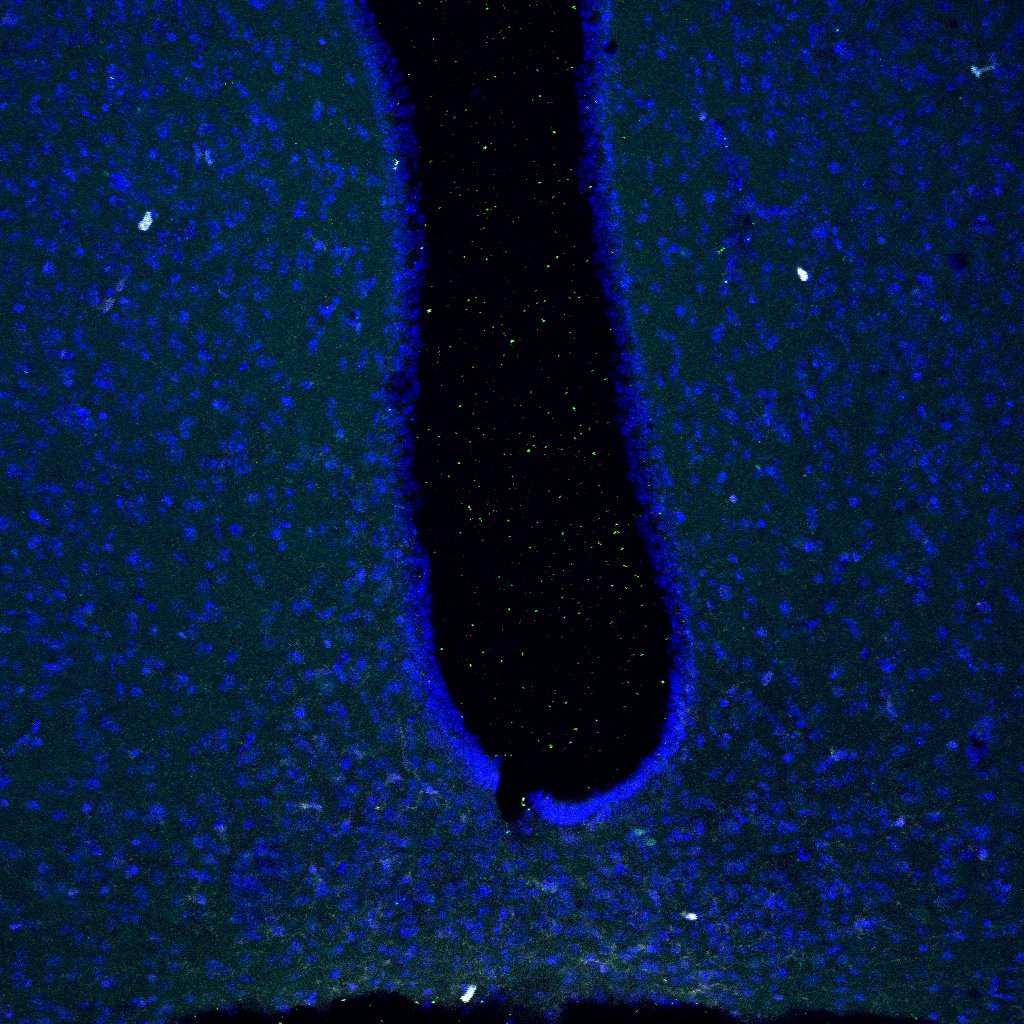

Supplement: Supplementary file 4 — Source Data Fig. 2 [file 44319_2023_16_MOESM4_ESM.zip › Fig2P picture.jpg]

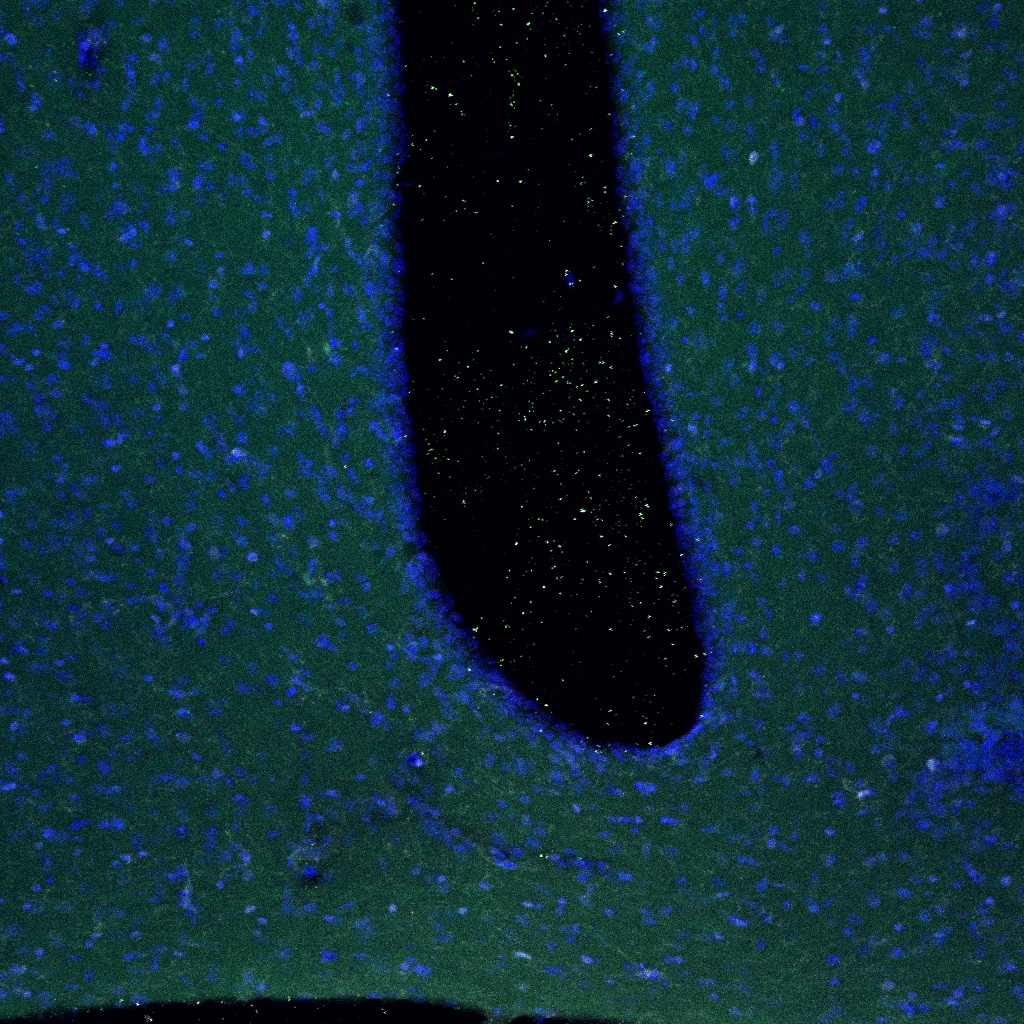

Supplement: Supplementary file 4 — Source Data Fig. 2 [file 44319_2023_16_MOESM4_ESM.zip › Fig2Q picture.jpg]

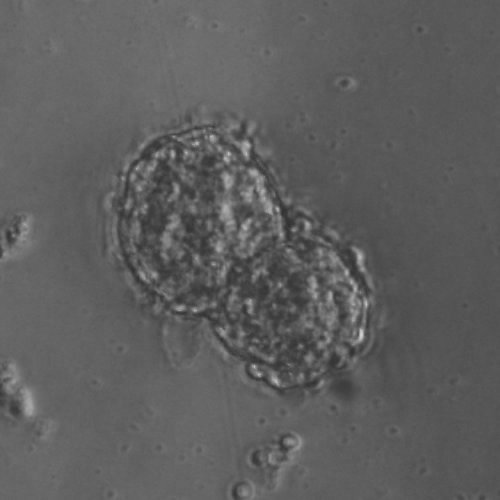

Supplement: Supplementary file 5 — Source Data Fig. 3 [file 44319_2023_16_MOESM5_ESM.zip › Fig3A picture.jpg]

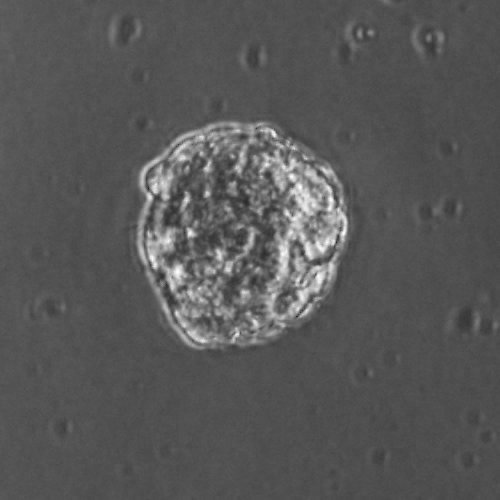

Supplement: Supplementary file 5 — Source Data Fig. 3 [file 44319_2023_16_MOESM5_ESM.zip › Fig3B picture.jpg]

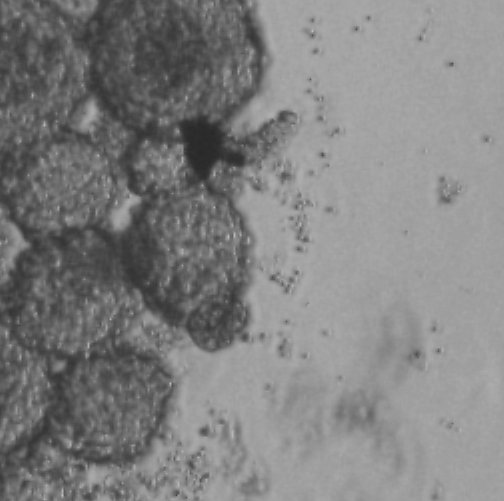

Supplement: Supplementary file 5 — Source Data Fig. 3 [file 44319_2023_16_MOESM5_ESM.zip › Fig3C picture.jpg]

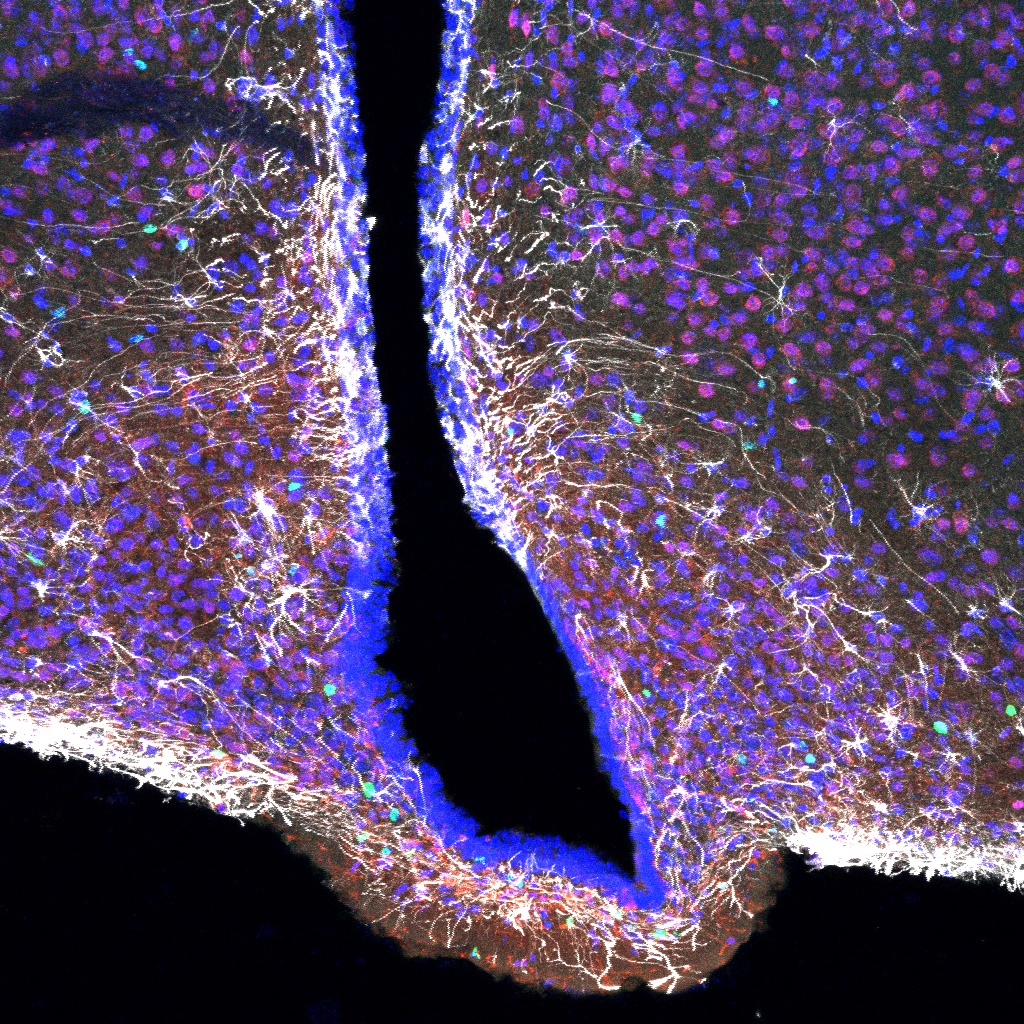

Supplement: Supplementary file 6 — Source Data Fig. 4 [file 44319_2023_16_MOESM6_ESM.zip › Fig4A picture.jpg]

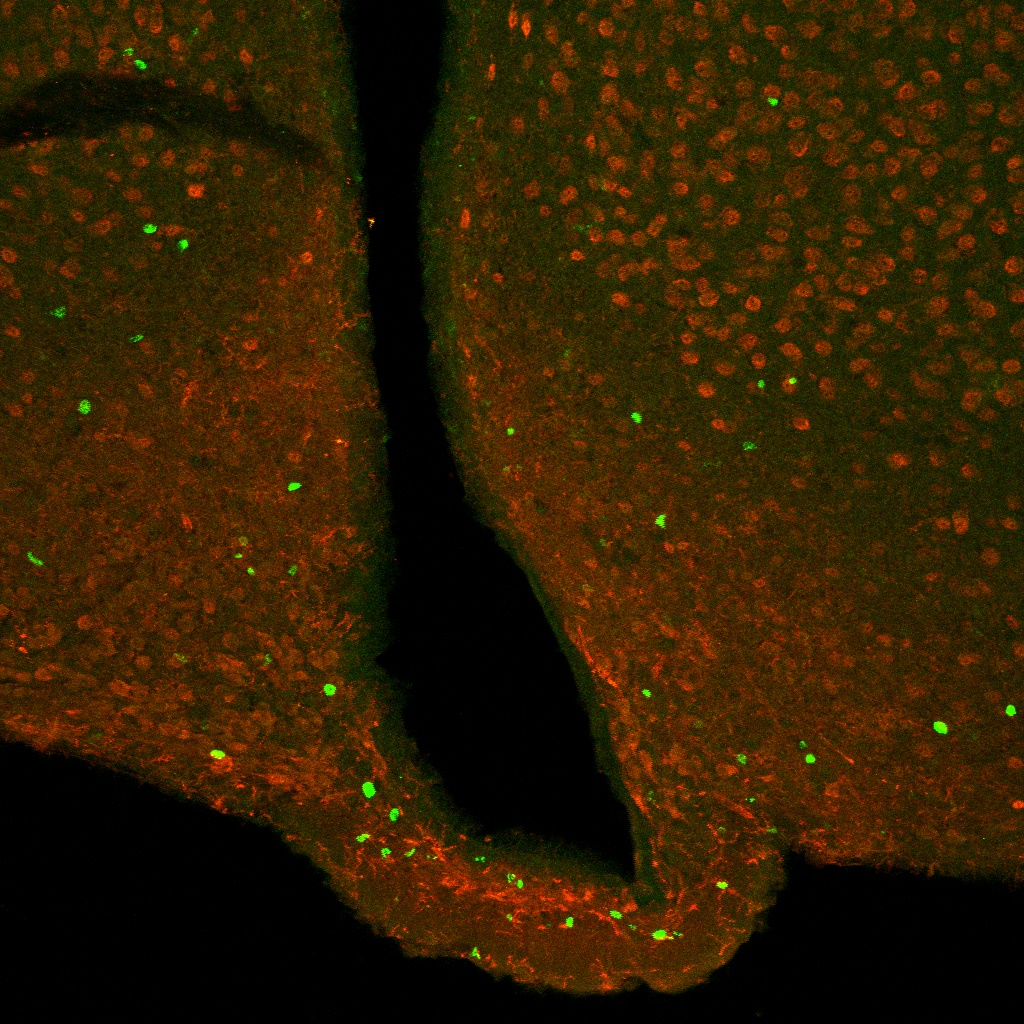

Supplement: Supplementary file 6 — Source Data Fig. 4 [file 44319_2023_16_MOESM6_ESM.zip › Fig4A' picture.jpg]

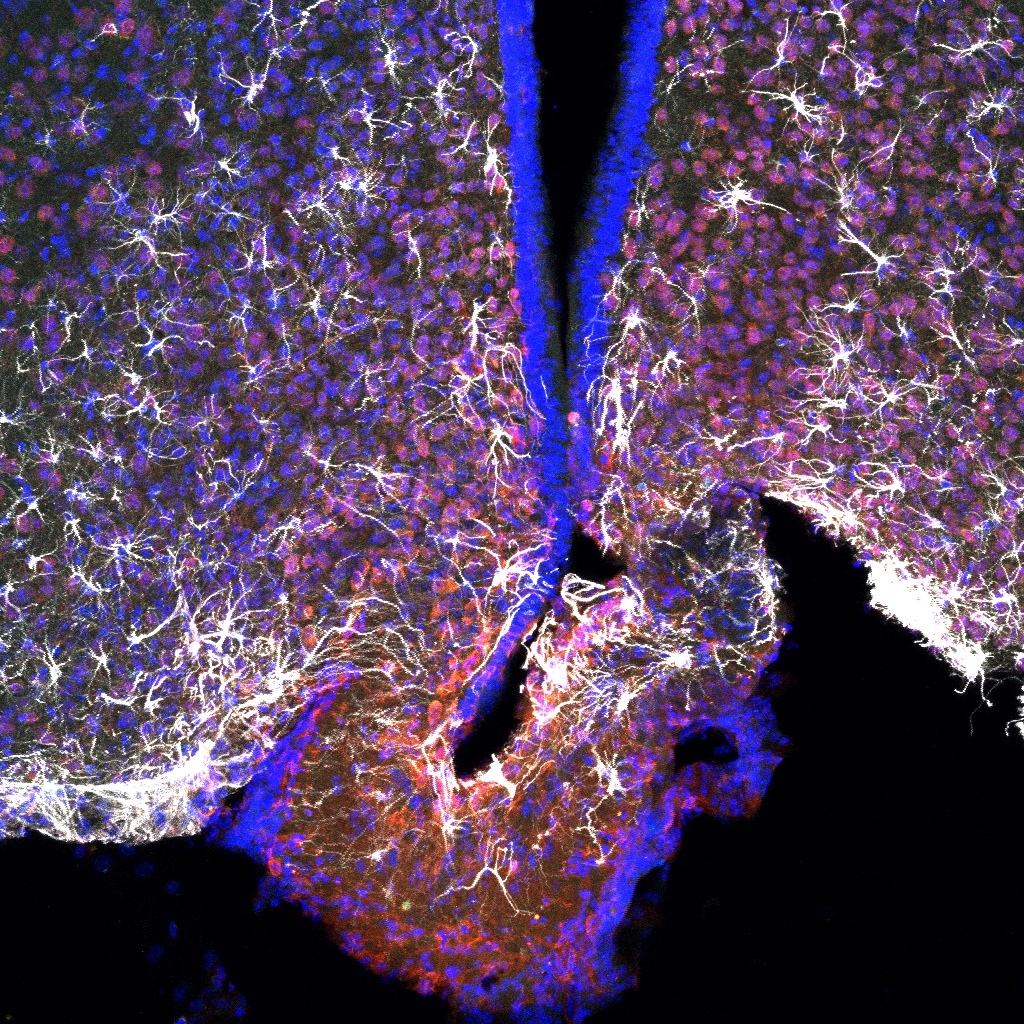

Supplement: Supplementary file 6 — Source Data Fig. 4 [file 44319_2023_16_MOESM6_ESM.zip › Fig4B picture.jpg]

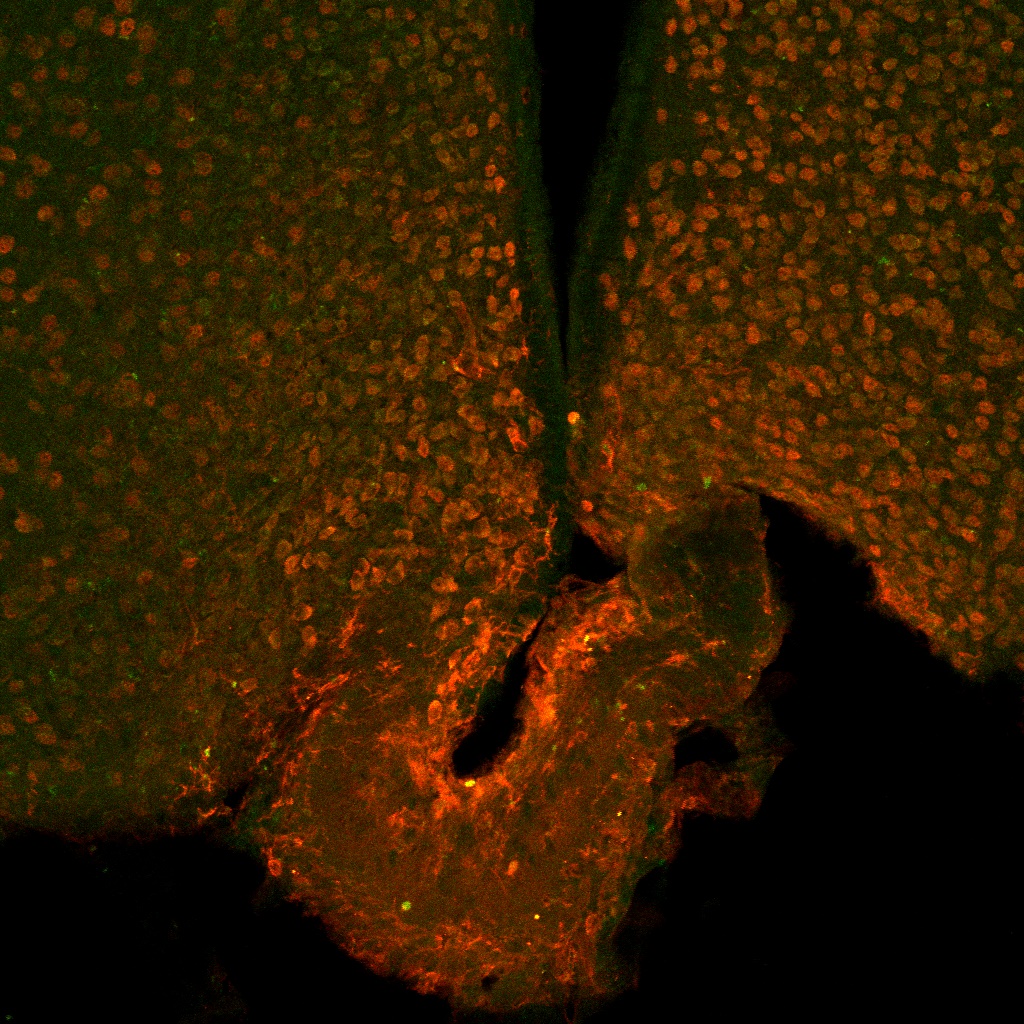

Supplement: Supplementary file 6 — Source Data Fig. 4 [file 44319_2023_16_MOESM6_ESM.zip › Fig4B' picture.jpg]

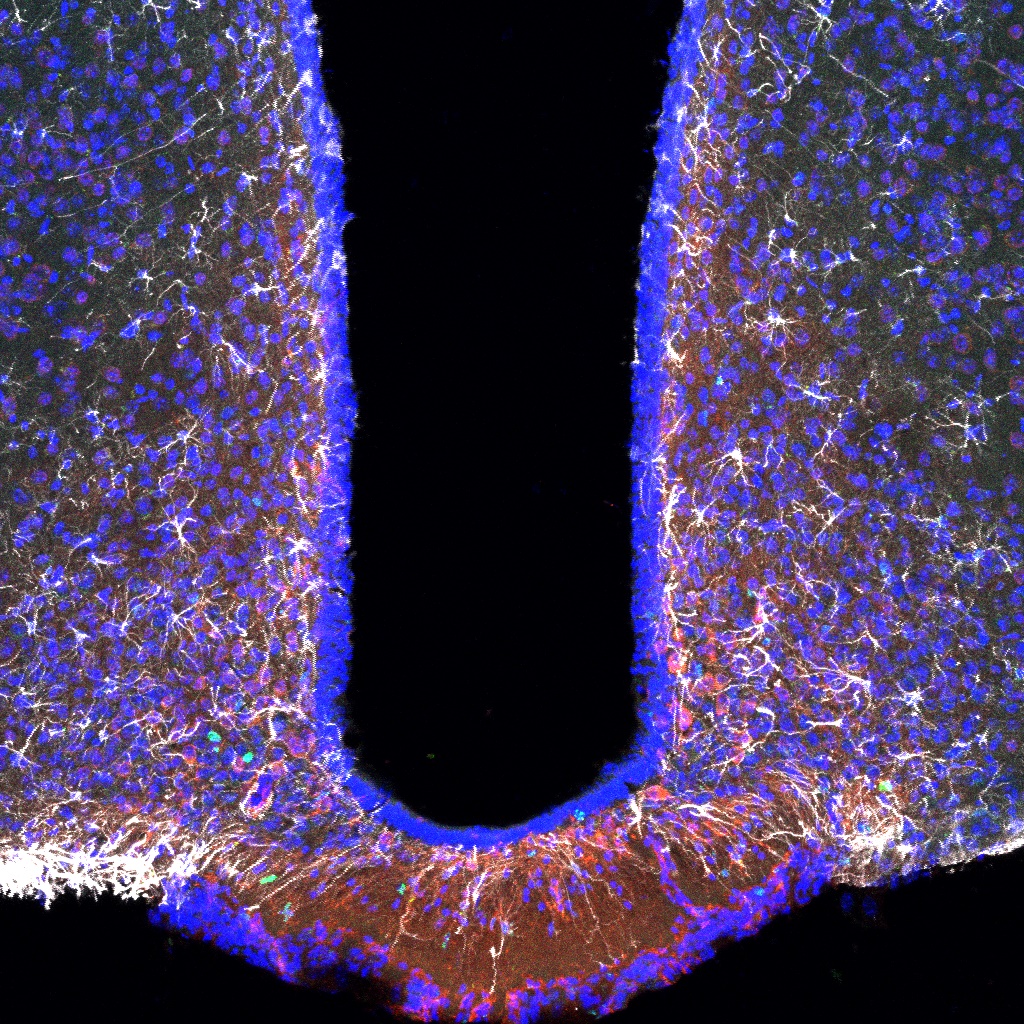

Supplement: Supplementary file 6 — Source Data Fig. 4 [file 44319_2023_16_MOESM6_ESM.zip › Fig4C picture.jpg]

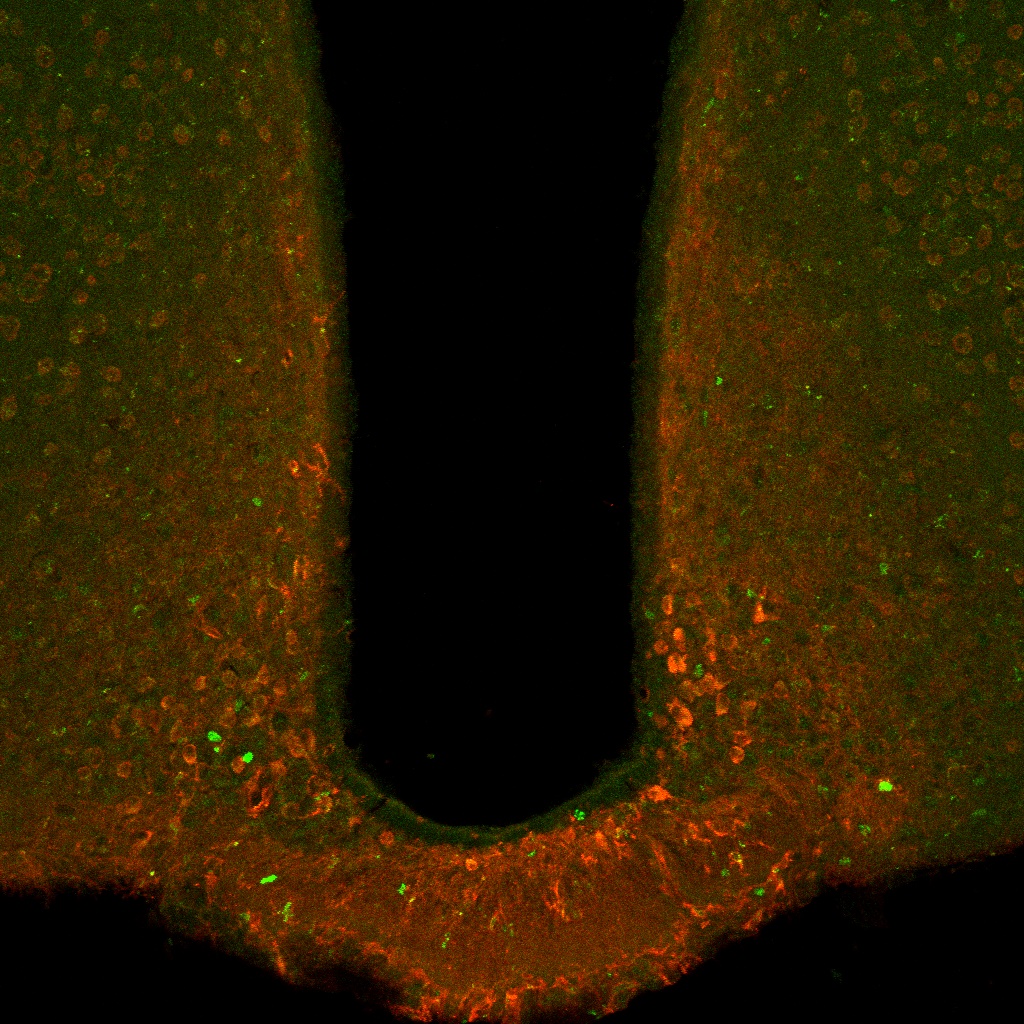

Supplement: Supplementary file 6 — Source Data Fig. 4 [file 44319_2023_16_MOESM6_ESM.zip › Fig4C' picture.jpg]

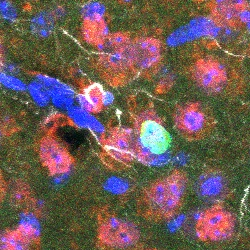

Supplement: Supplementary file 6 — Source Data Fig. 4 [file 44319_2023_16_MOESM6_ESM.zip › Fig4D picture.png]

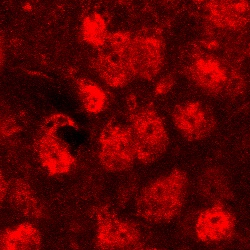

Supplement: Supplementary file 6 — Source Data Fig. 4 [file 44319_2023_16_MOESM6_ESM.zip › Fig4D' picture.png]

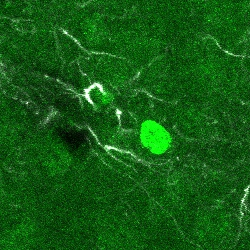

Supplement: Supplementary file 6 — Source Data Fig. 4 [file 44319_2023_16_MOESM6_ESM.zip › Fig4D'' picture.png]

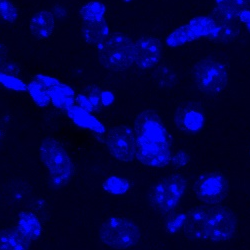

Supplement: Supplementary file 6 — Source Data Fig. 4 [file 44319_2023_16_MOESM6_ESM.zip › Fig4D''' picture.png]

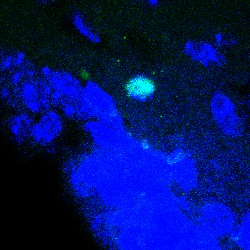

Supplement: Supplementary file 6 — Source Data Fig. 4 [file 44319_2023_16_MOESM6_ESM.zip › Fig4J picture.png]

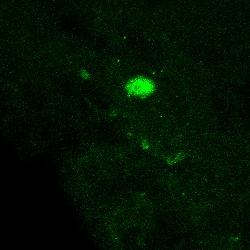

Supplement: Supplementary file 6 — Source Data Fig. 4 [file 44319_2023_16_MOESM6_ESM.zip › Fig4J' picture.png]

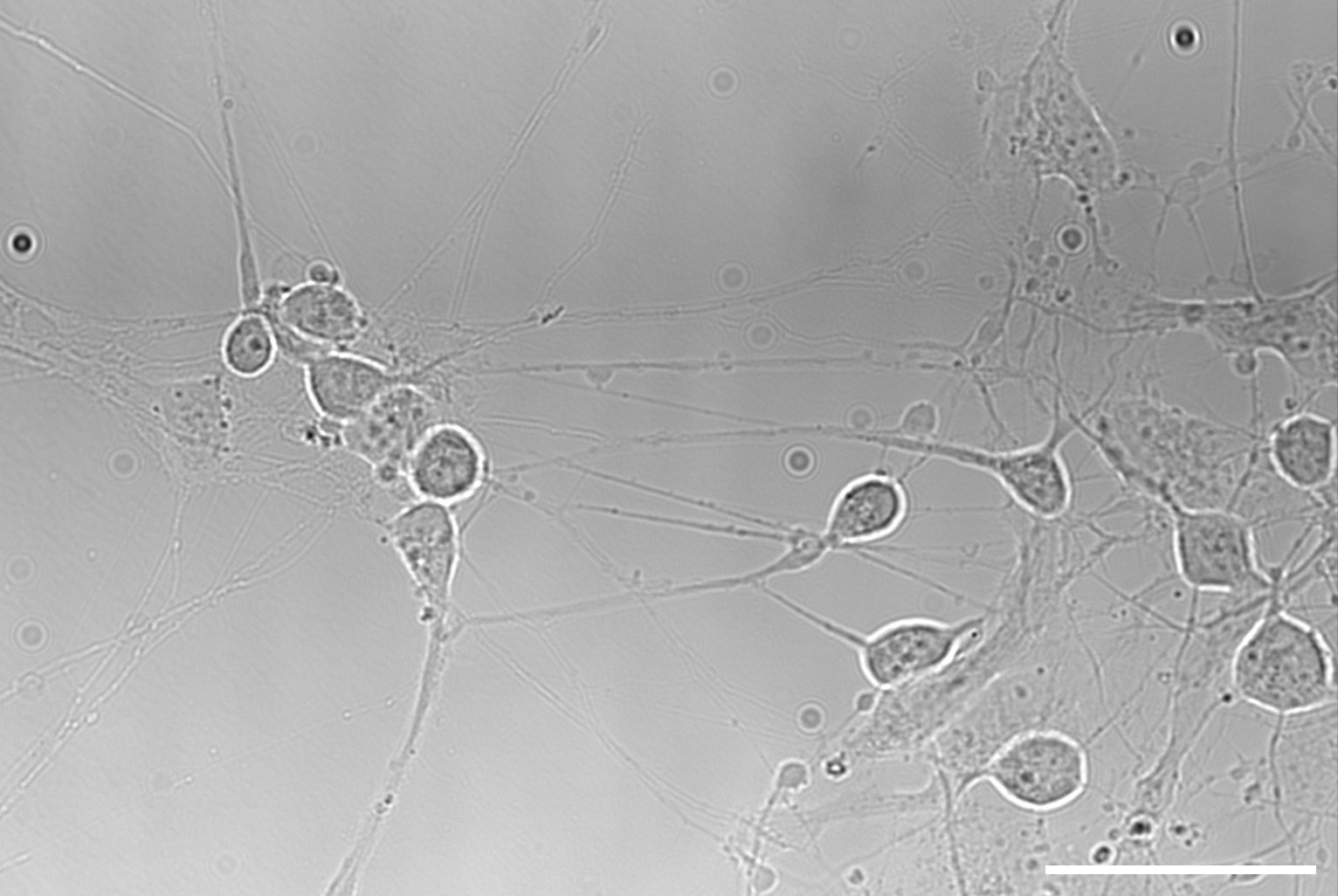

Supplement: Supplementary file 7 — Source Data Fig. 5 [file 44319_2023_16_MOESM7_ESM.zip › Fig5D picture.jpg]

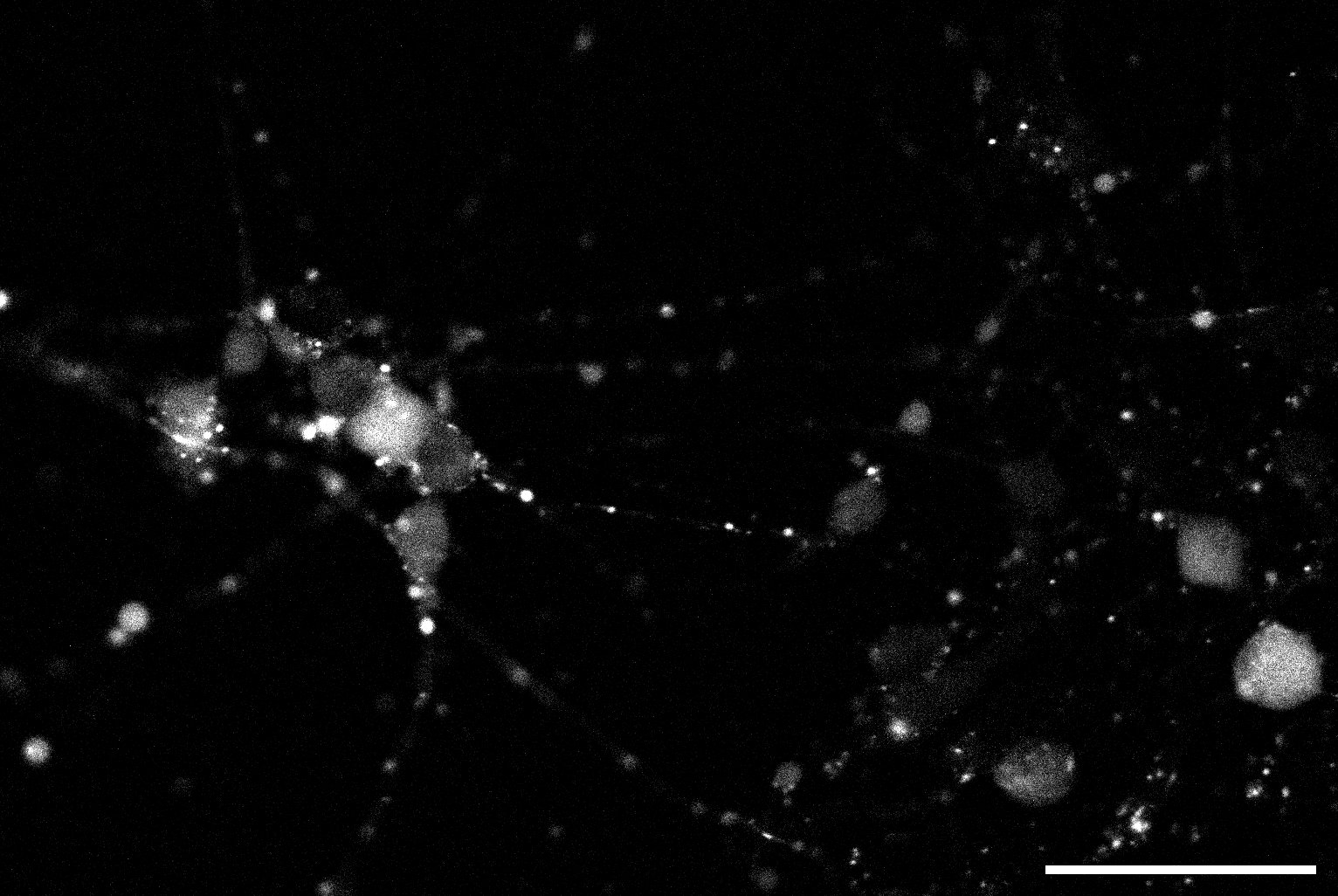

Supplement: Supplementary file 7 — Source Data Fig. 5 [file 44319_2023_16_MOESM7_ESM.zip › Fig5D' picture.jpg]
