# Supplementary material for: An analogue of the Prolactin Releasing Peptide reduces obesity and promotes adult neurogenesis
Source: EMBO Rep. 2023 Dec 20;25(1):351–77. doi: 10.1038/s44319-023-00016-2 (PMC10897398; doi:10.1038/s44319-023-00016-2)
Supplement: Supplementary file 8 — Expanded View Figures [file 44319_2023_16_MOESM8_ESM.pdf]

Expanded View Figures

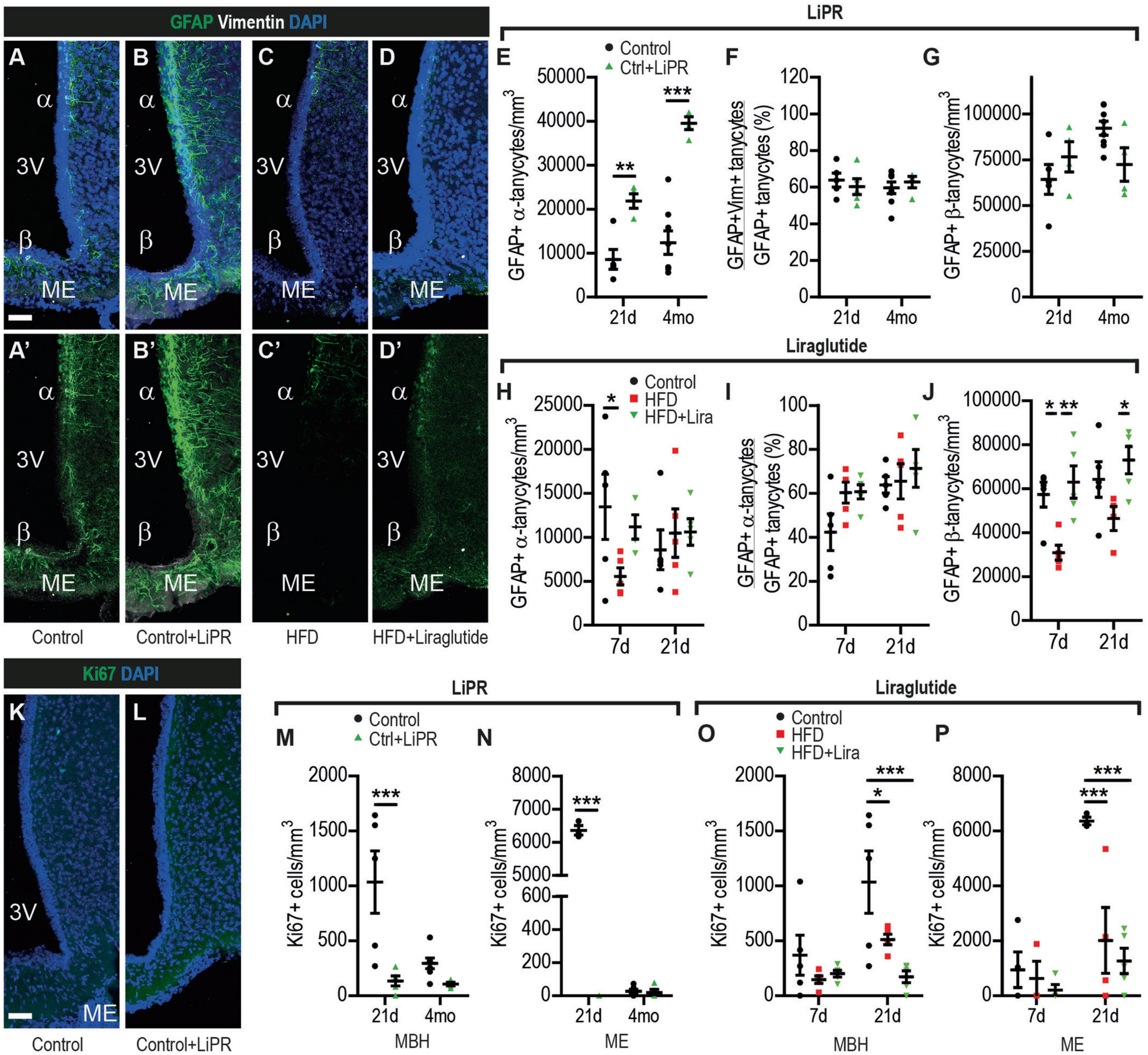

Figure EV1. Effects of LiPR in Control Diet and Liraglutide on tanycytes and proliferating cells in the MBH.

(**A–D**) Representative confocal images of HVZ stained as indicated in Control (**A**), Control+LiPR (**B**), HFD (**C**) and HFD + Liraglutide (Lira) (**D**) of 21d HFD group. Images in panels **A** and **C** are identical with representative confocal images in Fig. 2A and B and are shown for direct comparison with the control in both figures. (**E–G**) Effects of LiPR on number of GFAP+ α-tanycytes per volume of MBH (**E**), the proportion of GFAP+Vimentin+ tanycytes (**F**) and the number of GFAP+ β-tanycytes in MBH (**G**). (**H–J**) Effects of Liraglutide on GFAP+ α-tanycytes (**H**), the proportion of GFAP+Vimentin+ tanycytes (**I**) and the number of GFAP+ β-tanycytes in MBH (**J**). (**K,L**) Representative confocal images of MBH stained for Ki67 in Control (**K**) and Control+LiPR (**L**) of 4mo treatment group. (**M,N**) Quantification of Ki67+ cells in the MBH (M) and in the ME (**N**) from Control+LiPR mice. (**O,P**) Quantification of Ki67+ (**O**) and PCNA+ cells (**P**) in the MBH of Liraglutide-treated mice. Data information: Scale bars: 50 μm. $n = 5$ mice (7d and 21d), $n = 8$ mice (4mo Control), $n = 4$ mice (4mo Control + LiPR). In panel **P**, One-Way ANOVA. In all other panels, Two-Way ANOVA (**E**: treatment $F(1,17) = 59.74$, $p < 0.0001$, duration $F(1,17) = 16.87$, $p = 0.0007$, interaction $F(1,17) = 7.04$, $p = 0.017$; **J**: treatment $F(2,23) = 11.36$, $p = 0.0004$, duration $F(1,23) = 4.42$, $p = 0.047$; **M**: treatment $F(1,17) = 14.52$, $p = 0.0014$, duration $F(1,17) = 7.24$, $p = 0.016$, interaction $F(1,17) = 6.2$, $p = 0.023$; **N**: treatment $F(1,14) = 4481$, $p < 0.0001$, duration $F(1,14) = 4404$, $p < 0.0001$, interaction $F(1,14) = 6.2$, $p < 0.0001$; **O**: treatment $F(2,24) = 7.04$, $p < 0.0039$, duration $F(1,24) = 8.35$, $p < 0.0081$, interaction $F(2,24) = 3.01$, $p < 0.068$; **P**: treatment $F(2,17) = 10.11$, $p < 0.0013$, duration $F(1,17) = 21.99$, $p < 0.0002$, interaction $F(2,17) = 6.1$, $p < 0.01$). $^{*}p < 0.05$, $^{**}p < 0.01$, $^{***}p < 0.001$ (Bonferroni's test). Data are presented as mean ± SEM.

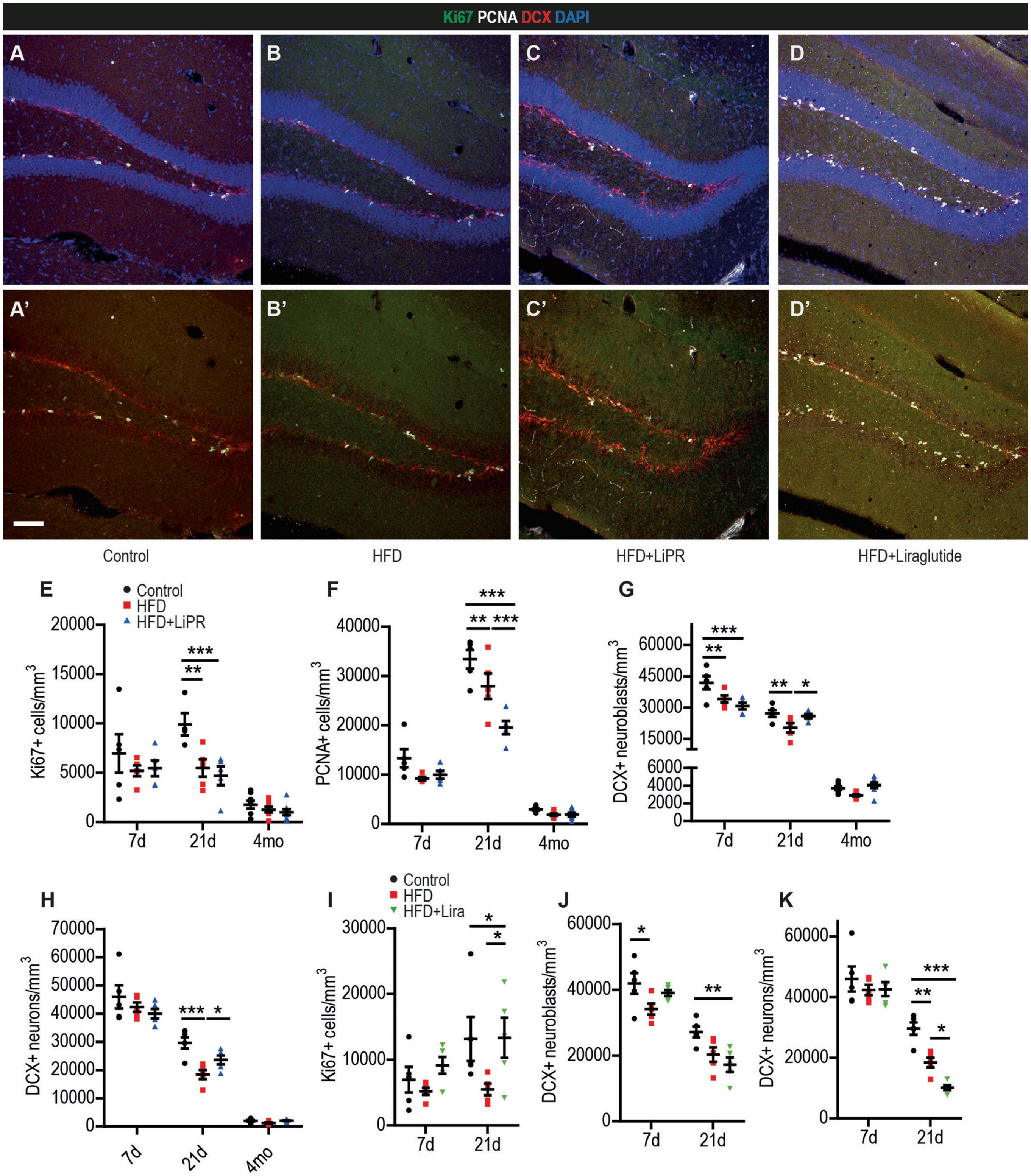
Ki67 PCNA DCX DAPI
A
B
C
D
A'
B'
C'
D'
Control
HFD
HFD+LiPR
HFD+Liraglutide
E
F
G
H
I
J
K
Control
HFD
HFD+LiPR
HFD+Lira
Ki67+ cells/mm3
PCNA+ cells/mm3
DCX+ neuroblasts/mm3
DCX+ neurons/mm3
7d
21d
4mo

Figure EV2. LiPR promotes neurogenesis in the SGZ.

(**A–D**) Representative confocal images of the Dentate Gyrus (DG) of the hippocampus showing the Subgranular Zone (SGZ) stained as indicated in Control (**A**), HFD (**B**), HFD + LiPR (**C**) and HFD + Lira of 21d HFD group. (**E–H**) Quantification of Ki67+ (**E**), PCNA+ cells (**F**), DCX+ neuroblasts (**G**) and DCX+ neurons (**H**) in SGZ for Control, HFD and HFD + LiPR. (**I–K**) Cell quantification in SGZ as described for Control, HFD and HFD + Liraglutide. Data information: Scale bars: 50 μm. $n = 5$ mice per data set for 7d and 21d groups, $n = 8$ mice per data set for 4mo group. All panels, Two-Way ANOVA (**E**: treatment $F(2,44) = 9.88$, $p = 0.0003$, duration $F(2,44) = 6.86$, $p = 0.0002$, interaction $F(4,44) = 9.88$, $p = 0.0003$; **F**: treatment $F(2,45) = 21.19$, $p < 0.0001$, duration $F(2,45) = 374.3$, interaction $F(4,45) = 9.88$, $p < 0.0001$; **G**: treatment $F(2,45) = 11.38$, $p < 0.0001$, duration $F(2,45) = 463.03$, $p < 0.0001$; interaction $F(4,45) = 6.25$, $p = 0.0004$; **H**: treatment $F(2,45) = 8.77$, $p = 0.0006$, duration $F(2,54) = 552.73$, $p < 0.0001$, interaction $F(4,45) = 3.78$, $p = 0.011$; **I**: treatment $F(2,24) = 4.3$, $p = 0.025$,duration $F(1,24) = 4.22$, $p = 0.05$; **J**: treatment $F(2,24) = 7.21$, $p = 0.0035$, duration $F(1,24) = 95.86$, $p < 0.0001$; **K** treatment $F(2,24) = 12.29$, $p = 0.0002$, duration $F(1,24) = 161.57$, $p < 0.0001$, interaction $F(2,24) = 5.96$, $p = 0.0079$). $^*p < 0.05$, $^{**}p < 0.01$, $^{***}p < 0.001$ (Bonferroni's test). Data are presented as mean ± SEM.

© The Author(s)

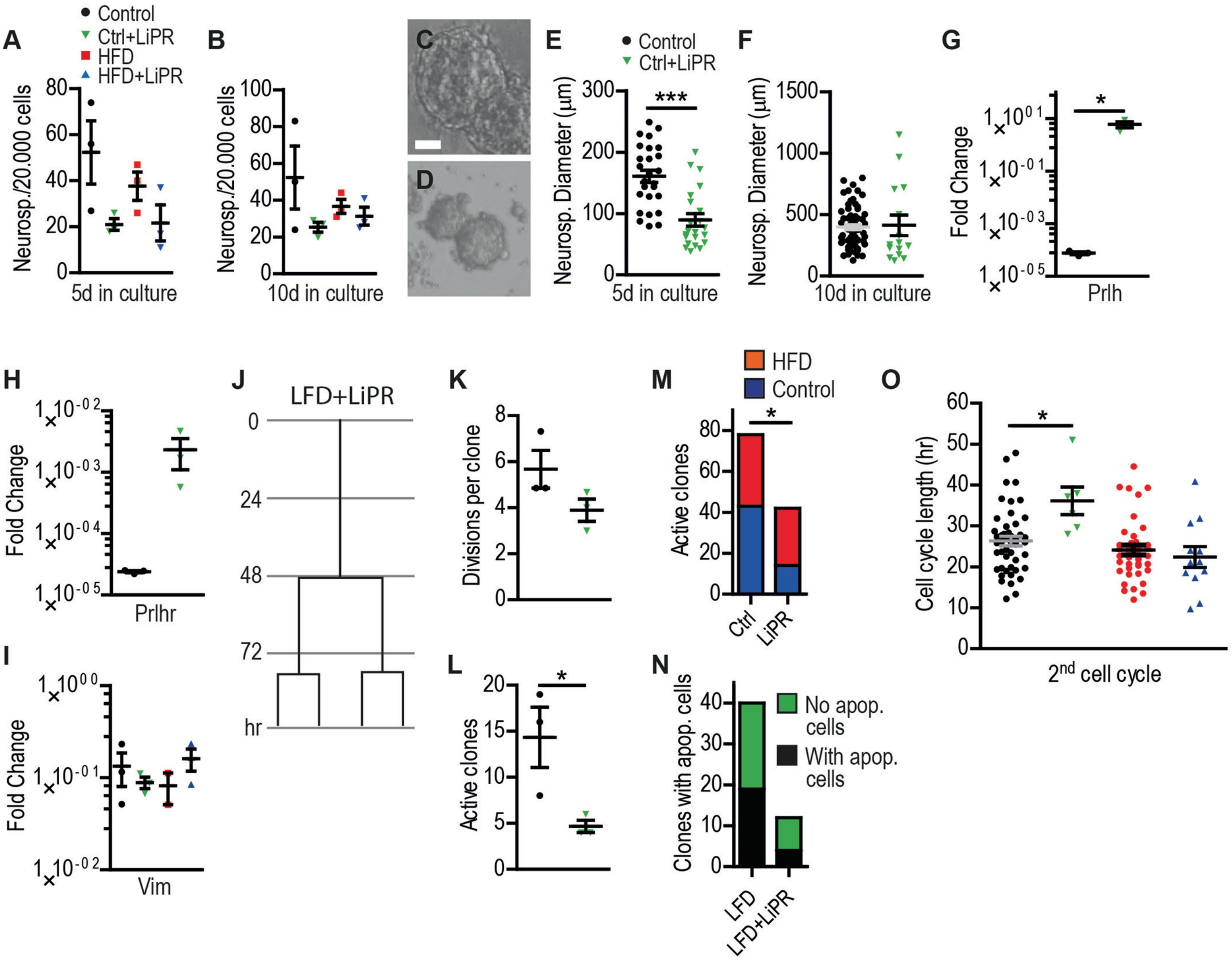

Figure EV3. LiPR reduces proliferation of naïve neurospheres.

(**A**,**B**) Quantification of number of HVZ-derived neurospheres per 20.000 plated cells after 5d (**A**) and 10d (**B**) in culture. (**C**,**D**) Representative images of HVZ-derived neurospheres 5d in culture from Control (**C**) and Control + LiPR (**D**) treated mice. The Image in panel **C** is identical with the image in Fig. 3A and is shown for direct comparison with the control in both figures. (**E**,**F**) Quantification of diameter of neurospheres 5d (**E**) and 10d (**F**) in culture. (**G-I**) Relative mRNA fold change of Prlh (**G**), Prlhr (**H**) and Vimentin (**I**) compared to Gapdh in cDNA from MBH-derived neurospheres (10d in culture). (**J**) An example cell division tree from 4-day time-lapse imaging of aNSCs from HVZ of Control + LiPR treated mouse. (**K**) Quantification of the number of cell divisions per division tree. (**L**) Time-lapse quantification of active (dividing) clones per 20.000 plated cells. (**M**) Number of all active clones of HVZ aNSCs from Control or LiPR-treated mice exposed to Control or HFD. Active clones pooled from all observed time-lapse imaging regions of interests per given treatment group. (**N**) Proportion of active clones containing at least one apoptotic cell. (**O**) Cell cycle length from the time-lapse imaging in the 2nd observed cell division. Data information: Scale bar: 20 μm. In all panels, $n = 3$ mice per data set. In panel **E**, number of neurospheres: $n = 26$ (Control), 22 (Control+LiPR). In panel **F**, number of neurospheres: $n = 66$ (Control), 15 (Control+LiPR). For panel **K**,**L**, number of traced cells: $n = 25$ per data set. In panel **O**, number of traced cells: $n = 47$ (Control), 6 (Control+LiPR), 35 (HFD), 12 (HFD+LiPR). Fisher's exact test (**M**,**N**), Un-paired two-tailed T-Test (**E–H**,**K**,**L**,**O**) and Two-Way ANOVA with Bonferroni's test (**A**,**B**,**I**). *$p < 0.05$, ***$p < 0.001$. Data are presented as mean ± SEM.

 © The Author(s)

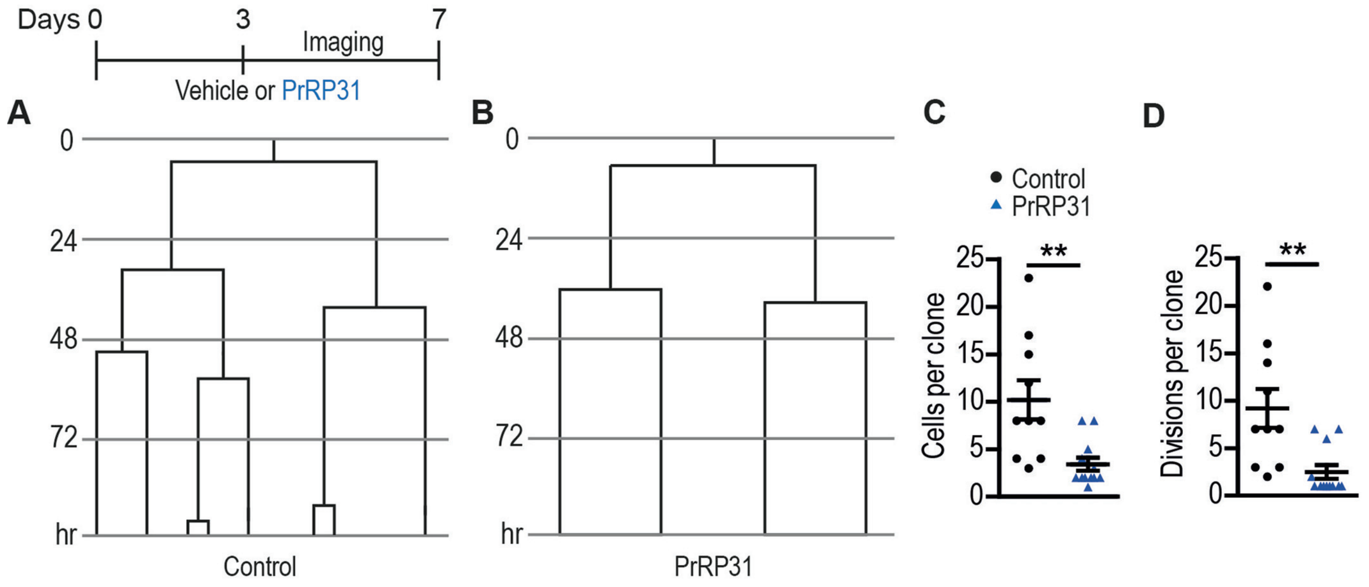

Figure EV4. LiPR reduces proliferation of naïve htNSCs.

(**A**,**B**) Example cell division trees from 4-day time-lapse imaging of aNSCs from HVZ of Control (**A**) and hPrRP31-treated (**B**) cells. A schematic of the experimental protocol shown above. (**C**,**D**) Quantification of cells per clone (**C**) and divisions per clone (**D**). Data information: In all panels, $n = 3$ mice per data set. In panels **C**,**D**, number of traced clones: $n = 10$ (Control), 12 (Control+LiPR). Un-paired two-tailed T-Test. $^{**}p < 0.01$. Data are presented as mean ± SEM.

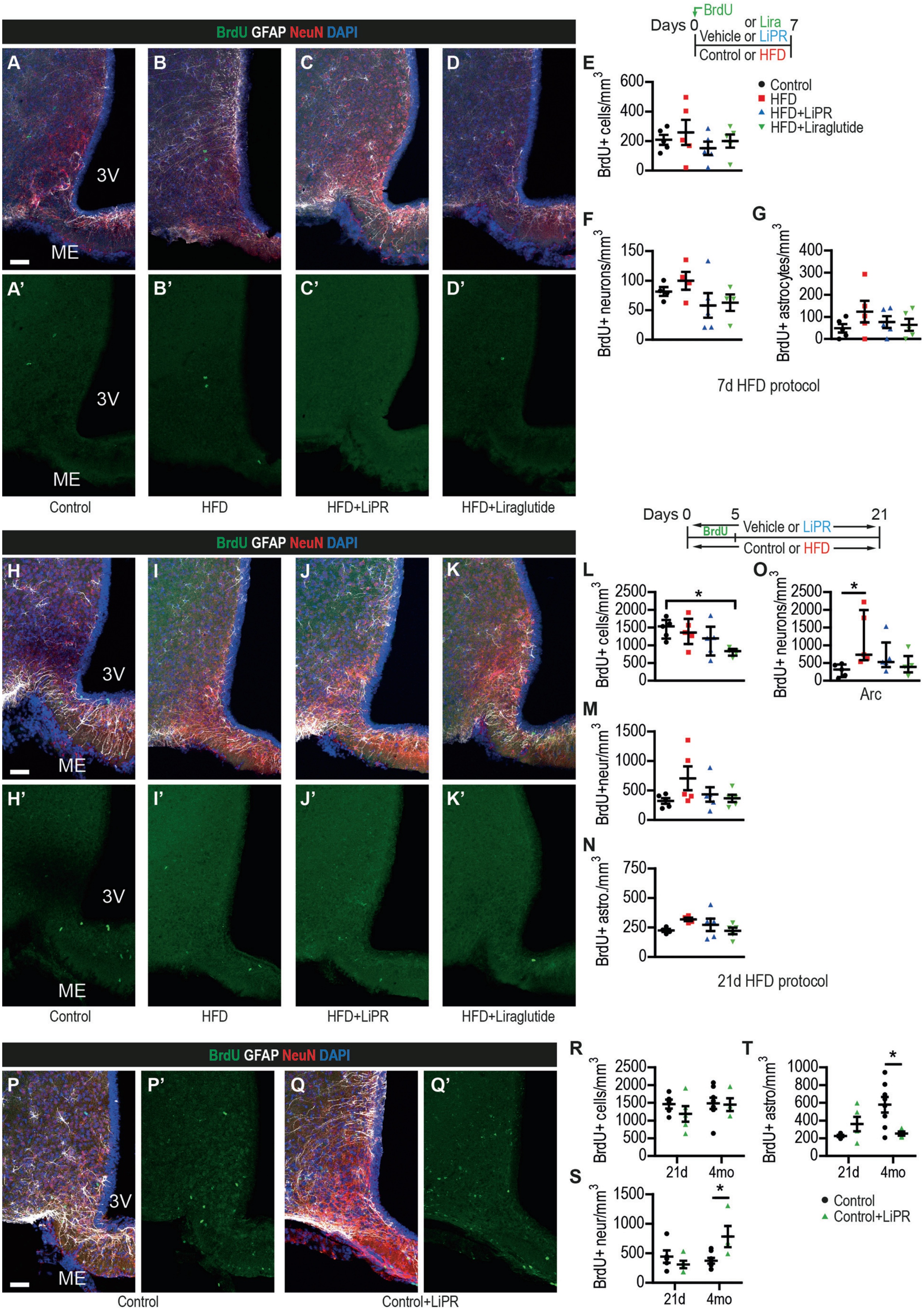
BrdU GFAP NeuN DAPI
A
B
C
D
3V
ME
A'
B'
C'
D'
Control
HFD
HFD+LiPR
HFD+Liraglutide
Days 0
BrdU
or Lira
7
Vehicle or LiPR
Control or HFD
E
BrdU+ cells/mm3
Control
HFD
HFD+LiPR
HFD+Liraglutide
F
BrdU+ neurons/mm3
G
BrdU+ astrocytes/mm3
7d HFD protocol
H
I
J
K
H'
I'
J'
K'
Days 0
5
21
BrdU
Vehicle or LiPR
Control or HFD
L
BrdU+ cells/mm3
O
BrdU+ neurons/mm3
Arc
M
BrdU+neur/mm3
N
BrdU+ astro./mm3
21d HFD protocol
P
P'
Q
Q'
Control
Control+LiPR
R
BrdU+ cells/mm3
21d
4mo
T
BrdU+ astro/mm3
S
BrdU+ neur/mm3
Control
Control+LiPR

 © The Author(s)

Figure EV5. Effects of LiPR on new adult-generated cells in shorter HFD protocols and in the context of Control Diet.

(**A–D**, **H–K**) Representative confocal images of HVZ and MBH stained as indicated in Control (**A**,**H**,**P**), HFD (**B**,**I**), HFD + LiPR (**C**,**J**), HFD + Liraglutide (Lira) (**D**,**K**) and Control Diet + LiPR (**Q**) of 7d (**A–D**), 21d (**H–K**) and 4mo (**P**,**Q**) groups. (**E–G**) Quantification of BrdU+ cells (**E**), BrdU+ neurons (**F**) and BrdU+ astrocytes (**G**) in the MBH of 7d HFD group. (**L–N**) Quantification of BrdU+ cells (**L**), BrdU+ neurons (**M**) and BrdU+ astrocytes (**N**) in the MBH parenchyma and BrdU+ neurons in the Arc (**O**) of 21d HFD group. (**R–T**) Quantification of BrdU+ cells (**R**), BrdU+ neurons (**S**) and BrdU+ astrocytes (**T**) in MBH parenchyma of Control and Control Diet + LiPR treated mice of 21d and 4mo groups. Data information: Scale bars: 50 µm. $n = 5$ mice per data set (7d, 21d), $n = 8$ mice (Control 4mo), $n = 4$ mice (Control + LiPR 4mo) per data set. In panel **O**, Kruskal–Wallis test with Dunn's test ($H = 9.88$, $p = 0.02$). In panels **E**–G and **L**–N, One-Way ANOVA with Tukey's test (**L**: $F(3,19) = 3.58$, $p = 0.038$) or Bonferroni's test (**N**: $F(3,19) = 4.3$, $p = 0.021$). In panels **R**–**T**, Two-Way ANOVA with Bonferroni's test (**S**: treatment $F(1,18) = 5.71$, $p = 0.021$; duration $F(1,18) = 9.9$, $p = 0.0056$; interaction $F(1,18) = 6.43$, $p = 0.02$; **T**: treatment $F(1,17) = 1.32$, $p = 0.27$; duration $F(1,17) = 2.2$, $p = 0.15$; interaction $F(1,17) = 7.75$, $p = 0.013$). $^{*}p < 0.05$, $^{***}p < 0.001$. Data are presented as median ± SEM (**O**) or mean ± SEM (all other).

© The Author(s)
